# Illuminating Rembrandt’s *Chiaroscuro* in *The Night Watch*: the painting process of Van Ruytenburch’s costume

**DOI:** 10.1038/s40494-025-01874-w

**Published:** 2025-08-18

**Authors:** Nouchka De Keyser, Annelies van Loon, Francesca Gabrieli, Frederik Vanmeert, Fréderique T. H. Broers, Petria Noble, Steven De Meyer, Arthur Gestels, Victor Gonzalez, Mitra Almasian, Inez van der Werf, Erma Hermens, Koen Janssens, Katrien Keune

**Affiliations:** 1 Van’t Hoff Institute for Molecular Sciences, University of Amsterdam, Amsterdam, The Netherlands; 2 Conservation & Science, Rijksmuseum, Amsterdam, The Netherlands; 3 AXIS, Department of Physics, University of Antwerp, Antwerp, Belgium; 4 ARCHES, Faculty of Design Sciences, University of Antwerp, Antwerp, Belgium; 5 Collection and Science, Mauritshuis, The Hague, The Netherlands; 6 Inorganic Chemistry and Catalysis, Institute for Sustainable and Circular Chemistry, Utrecht University, Utrecht, The Netherlands; 7 Paintings Laboratory, Royal Institute for Cultural Heritage (KIK-IRPA), Brussels, Belgium; 8 Research Group InViLab, Department Electromechanics, University of Antwerp, Antwerp, Belgium; 9 PPSM CNRS/ENS Paris-Saclay, Université Paris-Saclay, Gif-sur-Yvette, France; 10 Biomedical Engineering and Physics, Amsterdam UMC, University of Amsterdam, Amsterdam, The Netherlands; 11 Cultural Heritage Agency of the Netherlands, Amsterdam, The Netherlands; 12 Hamilton-Kerr Institute and Conservation and Science Division, Fitzwilliam Museum, University of Cambridge, Cambridge, UK

## Abstract

This study examines Rembrandt’s use of *chiaroscuro* to depict the costume of Lieutenant Willem Van Ruytenburch, a prominently lit figure in *The Night Watch* (1642). As part of *Operation Night Watch*, the painting was analyzed using noninvasive imaging techniques, including reflectance imaging spectroscopy (RIS), macroscopic X-ray powder diffraction (MA-XRPD), and macroscopic X-ray fluorescence (MA-XRF). These methods enabled the mapping of the artist’s pigment palette, which includes lead white, lead-tin yellow, ochres, vermilion, arsenic sulfide pigments, red lakes, smalt, and azurite. Rembrandt applied these pigments in a consistent, systematic way, combining them in groups to achieve pictorial unity. Notably, arsenic-based pigments were used to capture the warm reflections of gold threads, unique to Van Ruytenburch’s costume. MA-XRPD also identified degradation products—mimetite, weddellite, and palmierite—associated with the original pigments. These results provide new insights into Rembrandt’s modus operandi and inform understanding of the current condition and implications for its conservation.

## Introduction

Rembrandt van Rijn (1606–1669) is arguably the most prominent painter of the Dutch seventeenth century and was held in high esteem by his contemporaries for his painterly skills. One of the defining characteristics of his work is his skillful manipulation of light and shadow, the so-called *chiaroscuro* painting technique^1^. *Chiaroscuro* refers to the use of strong contrasts between light and dark to create pictorial unity and drama in the composition^2^. Rembrandt was a master at employing *chiaroscuro*, not only to create convincing spatial illusion in his compositions but also to convey the narrative and atmosphere in his paintings. This not only allows the viewer to grasp the essence of the story at a single glance but also involves the viewer as one of the bystanders. Rembrandt accomplished this remarkably in 1642 with his first and only civic guard piece, *Officers and other civic guardsmen of District II in Amsterdam, under the command of Captain Frans Banninck Cocq and Lieutenant Willem van Ruytenburch*, known as *The Night Watch*.

The composition of *The Night Watch*, and especially Rembrandt’s use of *chiaroscuro* was already praised in his time by critics and artists alike and is still to this day extensively discussed by scholars^2–7^. Joachim von Sandrart (1606–1688), a German painter, praised Rembrandt when explaining the concept of *houding*: “the extremely brilliant Rembrandt: he has practically worked miracles […], and has consistently achieved true harmony with all the colors, without exception, in accordance with the rules of light.”^2,4^. Rembrandt’s pupil Samuel Dirksz. van Hoogstraten (1627–1678) complemented him specifically for the pictorial unity in *The Night Watch*^3^.

**Fig. 1:**
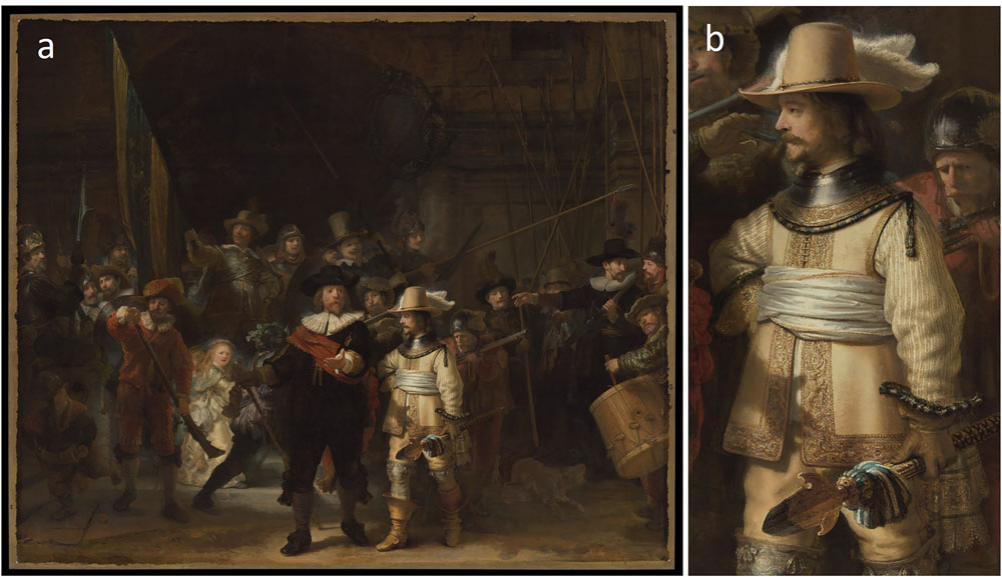
Lieutenant Willem van Ruytenburch's costume in *The Night Watch*. **a** Rembrandt van Rijn, *Militia Company of District II under the Command of Captain Frans Banninck Cocq*, known as *The Night Watch*, 1642, oil on canvas (wax-resin lined), 378.4 cm by 453.0 cm, Rijksmuseum, Amsterdam. **b** Detail showing Lieutenant Willem van Ruytenburch, one of the central figures in the composition.

This article discusses Rembrandt’s use of *chiaroscuro* in the depiction of materials in Lieutenant Willem van Ruytenburch’s costume in *The Night Watch* (Fig. 1). Our research aimed to provide a deeper understanding of the painting techniques that contributed to the texture of the materials in Van Ruytenburch’s costume, as well as an analysis of how Rembrandt planned and executed his light and shadow effects. Additionally, we explore how the original textures intended by Rembrandt have changed over time due to paint degradation processes.

The costume of Van Ruytenburch (Fig. 1b) was chosen as a focus for this study, as he is one of the most illuminated and key figures in the foreground of the composition. He wears yellow leather breeches and a sleeveless jerkin—a collarless, close-fitting hip-length jacket—accentuated by a golden embroidered border, layered over a doublet with yellow and white striped sleeves. His ensemble is completed with knee-height spurred riding boots, an ornate metal collar known as a gorget, a bejeweled plumed hat, and yellow leather gloves edged with luxurious yellow and blue fringes. Emblematic of his rank as a lieutenant, he holds a broad-bladed spear, known as a partisan, which is decorated with a blue and yellow tassel, the symbolic colors of the arquebusiers' company. Flanked on his proper right by Captain Frans Banninck Cocq, dressed in black, Van Ruytenburch plays an important role in the symbolic message of the composition as a whole, with the shadow of Banninck Cocq’s outstretched hand highlighting a detail in the embroidery, a lion holding the crowned arms of the city of Amsterdam (featuring three black St. Andrew’s crosses)^8^. In the service of spatial illusion, eminent scholar, Egbert Haverkamp-Begeman^9^ noted that Rembrandt rendered Van Ruytenburch’s costume using a wealth of detail, thickly textured *impasto* and color contrasts as compared to many of the figures positioned in the background, which are submerged into the shadows according to the aerial perspective. Aerial or atmospheric perspective is an important concept in art that describes how the atmosphere influences the appearance of objects viewed from a distance. By manipulating color, detail, and contrast, painters can guide the viewers’ eyes through a scene and evoke an illusion of depth and space^10^. By means of eye-movement tracking, De Winter et al. examined how people distribute their attention while observing *The Night Watch*. This revealed that Van Ruytenburch’s attire attracted much attention^11^, consistent with the notion that Rembrandt used light and texture to catch the attention of the viewer.

In the context of *Operation Night Watch*, a large-scale research and conservation project initiated in July 2019, the painting has been meticulously studied with the latest and most advanced analytical techniques on the macro- and microscale. One of the overarching goals of the research project, aside from assessing the condition of the painting and determining a treatment plan to ensure its future preservation, is to gain deeper insight into Rembrandt’s modus operandi, which this research aimed to explore. Several noninvasive imaging modalities, including macroscopic X-ray fluorescence imaging (MA-XRF), reflectance imaging spectroscopy in the visible to near-infrared spectral range (VNIR, 400–1000 nm) and the short wavelength infrared (SWIR, 900–2500 nm)^12^, macroscopic X-ray powder diffraction imaging (MA-XRPD) were performed to obtain spectral information on the macroscale.

Prior to *Operation Night Watch*, the painting technique of *The Night Watch* was studied in the 1970s using traditional imaging techniques, infrared photography and X-radiography, in addition to paint sample analysis^13–16^. The latter gives specific stratigraphic information and the identification of the pigments used by Rembrandt. Infrared photography and X-radiography gave insight into changes made by the artist and the painting process, as well as the distribution of radio-opaque pigments. However, these techniques are unable to provide a complete understanding of Rembrandt’s painting technique and the steps undertaken by the painter in the creative process.

Chemical imaging modalities, in particular, MA-XRF, MA-XRPD and reflectance imaging spectroscopy (RIS) have proven to be invaluable analytical methods since their development and application in the heritage field. These methods allow mapping of the artist’s pigment palette and provide insight into the painting techniques, steps in the painting process, and condition. Over the course of the last decade, artworks of many artists have been studied with these noninvasive methods, including Johannes Vermeer (1632–1675)^17–19^, Jan van Eyck (1390–1441)^20^, Rembrandt^21–25^, Michael Sweerts (1618–1664)^26,27^ and 17th-century still life painters such as Jan Davidsz. de Heem (1606–1684) and Abraham Mignon (1640–1679)^28–30^. These three imaging modalities are complementary and their combined use allows for a mutual compensation of the limitations of each method^20^. MA-XRF scanning provides elemental information on the surface and subsurface layers of the paint stratigraphy. RIS-VNIR detects spectral signatures from pigments that are predominantly present on the surface of the painting, based on the electronic transitions related to the color of materials^31^, while RIS-SWIR is sensitive to paint components located deeper in the paint stratigraphy and not directly associated with a visible color, such as the binding medium. MA-XRPD imaging allows mapping of crystalline pigment compounds on the paint surface and provides insight into crystalline degradation products that are present.

Supplemented with optical coherence tomography (OCT) measurements and micro-scale analysis of paint samples, these advanced imaging modalities provide the means to study the painting technique, pigment use and specifically Rembrandt’s sophisticated light-shadow modeling in *The Night Watch* on the macro- and microscale with high chemical specificity. OCT is an optical tomographic imaging modality that yields high-resolution, cross-sectional, subsurface images of the microstructure in materials by recording backscattered light^32^. The technique has been used in cultural heritage to capture high-resolution depth profiles of layered structures and study translucency and texture of certain paints; it was already applied to study the role of smalt in Rembrandt’s paints and the use of ground glass in red glazes^22,33^. To have complementary elemental and crystal structure information to the macroscale techniques, paint cross-sections were analyzed by means of scanning electron microscopy with energy dispersive X-ray spectroscopy (SEM-EDX) and synchrotron-based (SR-)XRPD imaging. Additionally, proteomics research was carried out with nano liquid chromatography-mass spectrometry (nano LC-MS) on two samples to ascertain the presence of proteinaceous material in impastos, which were previously found through high-pressure liquid chromatography (HPLC) by Karin Groen in 1997^15^. Nano LC-MS has proven to be more effective than HPLC for proteomics analysis due to its enhanced sensitivity, separation efficiency, and detailed analytical capabilities. It can handle complex mixtures, detect low-abundance proteins, and provide comprehensive proteome coverage with minimal sample requirements^34,35^.

The following paragraph introduces the concepts of *houding* and spatial illusion, which are central to understanding 17th-century painting techniques. In Baroque painting, spatial illusion and *houding* were important concepts to achieve pictorial unity. *Houding* might be translated in English as “attitude” and in essence refers to the tonal cohesion of the overall picture, where all the components have their correct place according to the proper light conditions. This methodology serves to approach reality as closely as possible and to ensure unity or an overall harmony in the composition, rather than present the viewer with a constellation of separate objects. Achieving pictorial unity and tonal cohesion, as well as a realistic rendering of the different materials in the composition, was a technical matter that numerous art theoretical and artist manuals devoted several chapters to, including Van Hoogstraten^3,36,37^. It demanded not just an understanding of material properties and material—light interaction for a convincing depiction and proper placement, but also a precise painting technique involving carefully calculated pigment mixtures from the ground up to the final glazes. According to Rembrandt scholar Ernst van de Wetering, for this reason, Rembrandt and his contemporaries worked systematically from the back to the front in pictorial space in such a way that each segment of the suggested reality in a painting was in principle painted with a specific palette, each containing the pigments necessary to create the intended illusion (incarnate, hair, textiles, etc)^38^. Various pictorial illusionistic effects to render specific surface textures can be found in contemporary literature, of which *De groote Waereld in ‘t kleen geschildert* or *The Big World Painted Small* (Amsterdam, 1962) by Dutch author Willem Beurs is among the most detailed, as he lists various recipes or paint instructions, including their application in lit and shaded parts, of various objects and materials such as metals (gold, silver, iron) to textiles of various colors from velvet, satin to silk.

Scholars have already noted Rembrandt’s use of illusionistic devices and pictorial tricks to manipulate the eye of the beholder, including his use of color and texture to position elements convincingly in a pictorial space. The general rule is that bright colors tend to come forward (especially warm ones, such as red and yellow), as do sharp tonal contrasts, such as the bright red vermilion in Banninck Cocq’s sash and the yellow of the girl’s dress and Van Ruytenburch’s costume. The placement of blue, purple, and green between the key figures helps create spatial recession in the composition.

Rembrandt’s use of texture in service of spatial illusion, described as the “rough matter” in the 17th century, has been discussed by many scholars in the past^39^. By using textured paint as visible brushstrokes or high impastos in the illuminated passages, while gradually leveling his paint towards smoother and more translucent paint mixtures in the shadowed areas, he manipulates the eye and the order in which objects are perceived. Although not directly referring to Rembrandt^40^, these optical illusionistic effects were described by contemporaries such as Du Fresnoy and de Piles and later by Rembrandt’s pupil Van Hoogstraten in a chapter titled “*Of Advancing, Receding, and Foreshortening”*, as the concept of “perceptibility or imperceptibility” (Dutch: “kenleykheyt of onkenlijkheyt”) or the effect of three-dimensionality^41^. The textured paint gives the eye something to focus on; it consequently appears closer to the viewer than the smoother painted passages that recede backward^2,3^.

A relevant passage for this research concerns a direct reference to Rembrandt’s technique made by Van Hoogstraten, in his *Inleyding tot de hooge schoole der schilderkonst: anders de zichtbaere werelt* (Introduction to the Academy of Painting; or: The Visible World), published in 1678. Van Hoogstraten notes in his fifth chapter on light and shadow and the gradations from light to dark that Rembrandt was a master in properly combining related colors and developed this virtue to a high degree when discussing the proper arrangement of light and shadows: Hoogstraten advises artists to follow Rembrandt’s example and not to overly blend lights and shadows, but rather to skillfully combine them in groups. He emphasizes the power of gentle accompaniment, suggesting that when strong lights are accompanied by lesser lights, their radiance is enhanced. Similarly, he highlights the importance of surrounding deepest darks with lighter darks to intensify the impact of light^3^. Rembrandt applied these principles directly in his work, using this approach to render *chiaroscuro*, in Van Ruytenburch’s costume, discussed in this article.

## Methods

Since July 2019, *Operation Night Watch* takes place in front of the public in the Gallery of Honor of the Rijksmuseum. A glass house (Meyvaert, Belgium) was constructed around *The Night Watch* ensuring enough working space to conduct research and movement of the painting as well as to maintain optimal visibility of the artwork for the public.

During the research phase, *The Night Watch* was unframed and mounted vertically on a custom-made easel (Bronnenberg, Heerlen) that is attached to a lead-bearing wall, enabling movements of the painting in the XYZ directions. Two scissor lifts (MTH Lifttechniek B.V., Alphen aan de Rijn) were installed in front of the painting, which enabled the researchers to safely study the whole surface of the painting. MA-XRF, MA-XRPD, stereomicroscopy and High-resolution 3D digital microscopy (Hirox) were performed on the scissor lifts. For other imaging equipment (RIS-VNIR, RIS-SWIR, 5 and 20 µm resolution photography), a custom-made imaging frame (Segula, now TBRM Engineering Solutions, NL) was used that consists of a vertically mounted 2D (x-y) gantry scanner with a scanning area of 5.4 × 4.4 m that covers the full surface area of the painting.

### Macro X-ray fluorescence (MA-XRF) imaging

Elemental mapping of *The Night Watch* was conducted using the commercially available MA-XRF scanner M6 Jetstream from Bruker Nano GmbH (Berlin, Germany). The M6 Jetstream comprises a measuring head with a 30 W Rh-target microfocus X-ray tube, a polycapillary lens, and dual 60 mm^2^ X-Flash silicon drift detectors with a beryllium window (offering an energy resolution <145 eV at Mn-Kα). This setup facilitated scanning the painting’s surface using an X, Y-motorized stage, covering an area of 80 × 60 cm^2^. The entire painting was scanned at 50 kV, with a current of 200 µA, utilizing a 500 µm step size and a dwell time of 35 ms.

A total of 56 scans, organized into eight rows and seven columns, with an ~20% overlap both horizontally and vertically, were necessary to fully scan the painting. The M6 Jetstream was securely mounted using a custom-designed rotating platform (Bronnenberg) on scissor lifts. To ensure a consistent instrument-to-painting distance (around 10 mm from the measurement head to the painting) and to maintain a 240 µm spot size while minimizing signal attenuation fluctuations in ambient air, meticulous positioning and alignment of the scanner were carried out before each scan.

Additionally, a detailed scan of the embroidered buff jerkin was performed at 50 kV, with a current of 200 µA, employing a 250 µm step size and a dwell time of 110 ms.

The resulting spectral data cubes of the MA-XRF scanning, shown in this paper, were processed using data analysis software packages PyMCA (Python Multichannel Analyzer) and Datamuncher gamma 1.4 and 1.5^42,43^. PyMCA was used to perform spectral fitting and deconvolution, in order to resolve overlapping peaks of different elements using the MCA Hypermet function. After spectral deconvolution, the separate elemental distribution images were obtained using the Datamuncher software. In the ensuing 2D distribution maps, each pixel carries information on the calculated net peak intensities of the emission lines of the element, with a gray scale linear to the detected intensities. The Co:As correlation plot was obtained with Datamuncher gamma 1.5.

### Macro X-ray powder diffraction (MA-XRPD) imaging

The MA-XRPD imaging measurements on *The Night Watch* were carried out using an in-house built mobile MA-XRF/XRPD scanning instrument from the AXIS research group (University of Antwerp), operating in reflection mode^44^. A monochromatic Cu-K_α_ (8.04 keV) X-ray source (50 W, Incoatec GmbH) was used with a photon flux of 2.9 × 10^8^, a focal diameter of ca. 140 µm, a focal distance of ca. 20 cm and a divergence of 2.4 mrad. An incident angle of 10° was chosen between the primary X-ray beam and the painting’s surface, leading to a beam with an elliptical footprint of ~1 × 0.2 mm^2^. Opposite the X-ray source, but on the same side as the painting, the PILATUS 200 K detector (Dectris Ltd., Switzerland) was positioned at an angle of ca. 40° with the painting surface to record the diffraction patterns. To ensure an equal distance between the X-ray source and the painting for every point of the scan, a laser distance sensor (Baumer Hold., CH) was used to automatically correct the position of the set-up for topographical variations. The aforementioned equipment is fastened to a motorized platform (motor stages from Newport Corp., USA) that allows moving in the XYZ planes.

The MA-XRPD set up was fixed on a spacer to securely fasten the equipment to the platform of the scissor lift. Due to the large size of the painting and time-consuming measuring time, several areas of interest were selected. The areas discussed in this paper are:

D06: Proper right shoulder of Lieutenant Van Ruytenburch. Analytical area (101.1 × 101.1 mm^2^) was mapped with a step size of 1.3 × 1.3 mm^2^ and a dwell time of 10 s pt^−1^.

D08: Bejeweled decoration around the brim of Van Ruytenburch’s hat. Analytical area (216.25 × 42.50 mm^2^) was mapped with a step size of 1.25 × 1.25 mm^2^ and a dwell time of 10 s pt^−1^.

D10-12: Embroidered border of Van Ruytenburch’s buff jerkin, at the height of Banning Cocq’s hand shadow casting. Analytical area (155.4 × 79.8 mm^2^) was mapped with a step size of 1.4 × 1.4 mm^2^ and a dwell time of 10 s pt^−1^.

The recorded XRPD data were processed using the in-house developed software package XRDUA from the AXIS research group (University of Antwerp)^45,46^. The obtained 2D distributions shown in this article are based on the global scaling factor obtained from the whole pattern fitting procedure.

### Reflectance imaging spectroscopy (Visible-to-near-infrared)

Visible-to-near-infrared (VNIR, 400–1000 nm) diffuse reflectance image cubes were acquired using a high-sensitivity hyperspectral camera (Surface Optics Corp, 710E model), equipped with a transmission grating-prism spectrometer combined with a backside illuminated EMCCD detector. The imaging camera has a spectral sampling of 2.5 nm, for a total of 260 channels and produces image cubes with 1024 × 1024 spatial pixels. The spatial resolution at the painting was 0.168 mm, corresponding to 172 mm field of view, and the integration time used was 100 ms. Two light sources Solux 4700 K, 50 W lamps (are coated to minimize UV and IR light) were used to collect the reflected signal. A step/stare collection approach was used to collect VNIR image cubes of the entire surface of the painting to allow generating a complete image cube. To do this, the camera and lights remained stationary, and the painting was moved left-right and up-down on an easel, and a total of 20 cubes (1024 × 1024 pixels) were acquired to have the final VNIR reflectance data cube. The conversion to apparent reflectance was done using a standard protocol, namely, flat fielding. A dark image cube (no light allowed into the camera) was collected along with an image cube of the illumination light reflected off a white diffuse reflectance standard (99% reflector, Labsphere, Inc.) that was placed in the plane of the painting. The apparent reflectance image cubes were calculated by dividing each raw cube collected of the painting after subtraction of the dark image cube by the image cube of the dark-subtracted diffuse reflectance standard. The 20 cubes were then stitched and registered to a visible image^47^. The derivative cube was calculated with MATLAB script from NGA and the endmembers were found manually in ENVI software (Harris Corp). Maps were made using spectral angle mapper algorithm (SAM) in the ENVI.

### 5 µm-resolution photography

To create this huge image, the painting was photographed in a grid with 97 rows and 87 columns with our 100 megapixel Hasselblad H6D 400 MS camera. Each of these 8439 separate photos, measuring 5.5 cm × 4.1 cm, was captured using a sophisticated laser-guided five-axis camera positioning system that can sense the precise location of the painting. Artificial intelligence was used to stitch these smaller photographs together to form the final large image, with a total file size of 5.6 terabytes^48^. https://www.rijksmuseum.nl/en/stories/operation-night-watch/story/ultra-high-resolution-photo.

### High-resolution 3D digital microscopy

The painted surface of *The Night Watch* was studied and photographed using a Hirox RH-2000 3D digital microscope on a motorized XY stage, equipped with a MXB-2500REZ lens. The microscope can achieve spatial sampling from 4.3 µm/pixel in Low-Range (35× magnification), 1.13 µm/pixel in Mid-range (140× magnification), and 0.45 µm/pixel in High Range (350x magnification). For the images in this paper, images were taken in Mid-Range. A white balance was made with a Color Checker white balance target of the Color Checker passport from X-Rite. The images were photographed under the same light conditions and exposure time.

### Optical coherence tomography (OCT)

OCT images were collected using a Thorlabs Telesto PS-OCT system, functioning at a center wavelength of 1300 nm, with a lateral and axial resolution of 13 and 5.5 μm in air, respectively. Volumetric images were collected covering a lateral area of 10 mm by 10 mm using 1024 × 1024 pixels, respectively. The OCT Images were post-processed using ImageJ version 1.54 f. Furthermore, layers of interest were segmented to obtain thickness and height maps using a custom-developed script in MATLAB version 2020a [Natick, Massachusetts: The MathWorks Inc.] in combination with Ilastik: interactive machine learning for (bio)image analysis version 1.3.2^33^. A refractive index of 1.5 was used for varnish to convert optical path length to physical distance in order to obtain the impasto height map (Fig. S1).

### Light microscopy

Cross-sections from 1975 to 1976 were reanalyzed for this article, and additional paint samples were taken for stratigraphic information or proteomic research. Two paint cross-sections, SK-C-5_016 and SK-C-5_017 were taken to identify and investigate the condition of the arsenic sulfide pigments. Sample SK-C-5_016 was taken in the proper right sleeve of Van Ruytenburch’s doublet in a warm brown stripe. Sample SK-C-5_017 was taken from the embroidered border of the sleeveless buff jerkin in an orange highlight on top of a brown underlayer. The paint samples were embedded in Poly-pol PS230: a two-component polyester mounting resin (Poly-Service Amsterdam, the Netherlands). SK-C-5_070 was taken in the yellow dress of the girl and embedded in Technovit 2000 LC mounting resin based on methacrylate, and cured under visible blue light (Heraeus Kulzer GmbH, Germany). The samples were polished using a sample holder and Micromesh sheets up to grade 12,000 (Micro-Surface Finishing Products Inc., Wilton, Iowa, USA)^49^. SK-C-5_1457 was taken in 1976 by R.M. Hesterman in the white feather of Van Ruytenburch’s hat.

Two paint cross-sections SK-C-5_089 and SK-C-5_090 were taken for nano LC-MS from the border of Van Ruytenburch’s jerkin at the proper left armpit in a damage from a lead white impasto (which includes part of the lead white underpaint) and a lead-tin yellow impasto, respectively (see Fig. S2 for sample locations).

All the cross-sections were photographed in bright field (BF) and dark field (DF) mode, and under ultraviolet radiation (UV365 nm) at spatial resolutions of 0.27 µm/pixel (200×) and 0.11 µm/pixel (500×) with a Zeiss Axio Imager. A2m microscope (Carl Zeiss Microscopy, LLC, United States) equipped with a Zeiss AxioCam 506 color digital camera. White light was provided by a LED lamp and a Colibri 2 controller was used for UV-fluorescence microscopy (LED light source at 365 nm, using a filter cube composed of a 365 nm excitation filter (EX G 365), a beam splitter at 395 nm (BS FT 395), and an emission long-pass filter at 420 nm (EM LP 420)). All images were obtained and processed in the image-acquisition software Zen 2 pro (blue edition) with extended depth of focus (MEDF) facilities and observed on a calibrated Eizo Color Edge CG277 BK computer screen.

### Scanning electron microscopy energy-dispersive X-ray spectroscopy (SEM-EDX)

Scanning electron microscopy studies in combination with energy dispersive X-ray spectroscopy were performed on a FEI NovaNano SEM 450 variable pressure electron microscope equipped with a Thermofisher NSS EDX system. The uncoated cross-sections were first analyzed in low vacuum and BSE images were taken with a GAD detector at an acceleration voltage of 15 kV, with a spot size of 3, at 90 Pa pressure and a 5 mm eucentric working distance. EDX spot analyses were done at a 6 mm working distance, with an acceleration voltage of 20 kV and spot size 3–5.

The samples were analyzed in high vacuum with a 4 nm thick tungsten coating to improve surface conductivity, using a Leica EM ACE600 Coater (8E-3 mbar). BSE-images were obtained with an accelerating voltage of 15 kV, spot 3, 6 mm working distance. EDX maps for SK-C-5_016 were also collected under high vacuum conditions, with an accelerating voltage of 20 kV, spot 5 and a map resolution of 512 × 340 pixels and a magnification of 650×.

### Synchrotron µ-XRPD-P06 beamline, PETRA III, DESY

Sample SK-C-5_016 was analyzed with SR-μ-XRPD at beamline P06, PETRA III, DESY. This hard X-ray micro- and nano-probe beamline is suited for X-ray powder diffraction imaging experiments at the (sub) micrometric scale. A Kirkpatrick-Baez optical system was used to focus the 21 keV beam to a diameter of 0.5 μm and a flux of ca. 10^10^ photons/s. The sample was mounted on a plastic frame that could be moved in the XYZ directions. An EIGER-X 4 M detector (Dectris Ltd., Switzerland) was used to collect the diffraction signals. The sample was placed 18 cm in front of the detector to achieve a sufficiently wide angular range.

An area of 150 × 270 µm^2^ was mapped using a step size of 1 µm in both directions. A dwell time of 0.25 s was used to acquire the diffraction patterns.

The in-house developed software package XRDUA was used for the processing of the XRPD data. The obtained 2D distributions shown in this article are based on the global scaling factor from the fitting procedure and the collected diffraction data were corrected for attenuation effects.

### Nano liquid chromatography mass spectrometry (LC-MS) for proteomics

To identify the presence of proteinaceous materials in *The Night Watch*, unembedded cross-section material SK-C-5_089 and SK-C-090 and various egg-oil lead white mixtures as reference samples were analyzed with nano LC-MS.

The egg-oil-lead white reference samples were prepared by Rosen Gramatikov in the context of his master's thesis titled: “Recreating Rembrandt’s Impastos and Understanding How Rembrandt Made His Paints”, University of Amsterdam, 2021. Table S1 lists the recipe of the paint references.

The samples were treated with PNGaseF followed by urea^50^. The extracted proteins were reduced and alkylated. Enzymatic hydrolysis was carried out with trypsin and the sample was desalted with a ZipTip C18 pipette tip^51^. The peptide mixtures were analyzed using nano liquid chromatography (Ultimate 3000, Thermo Scientific™) coupled with a Q Exactive™ Focus Hybrid Quadrupole-Orbitrap (Thermo Scientific™) via an EASY-Spray™ source. The MS/MS spectra were searched against Chordata in the SwissProt database using Mascot software version 2.6.2. Protein identifications were accepted with two or more peptides.

## Results and discussion

### Build-up of the embroidered buff jerkin and doublet: *textured lead white underpaint and smalt*

**Fig. 2:**
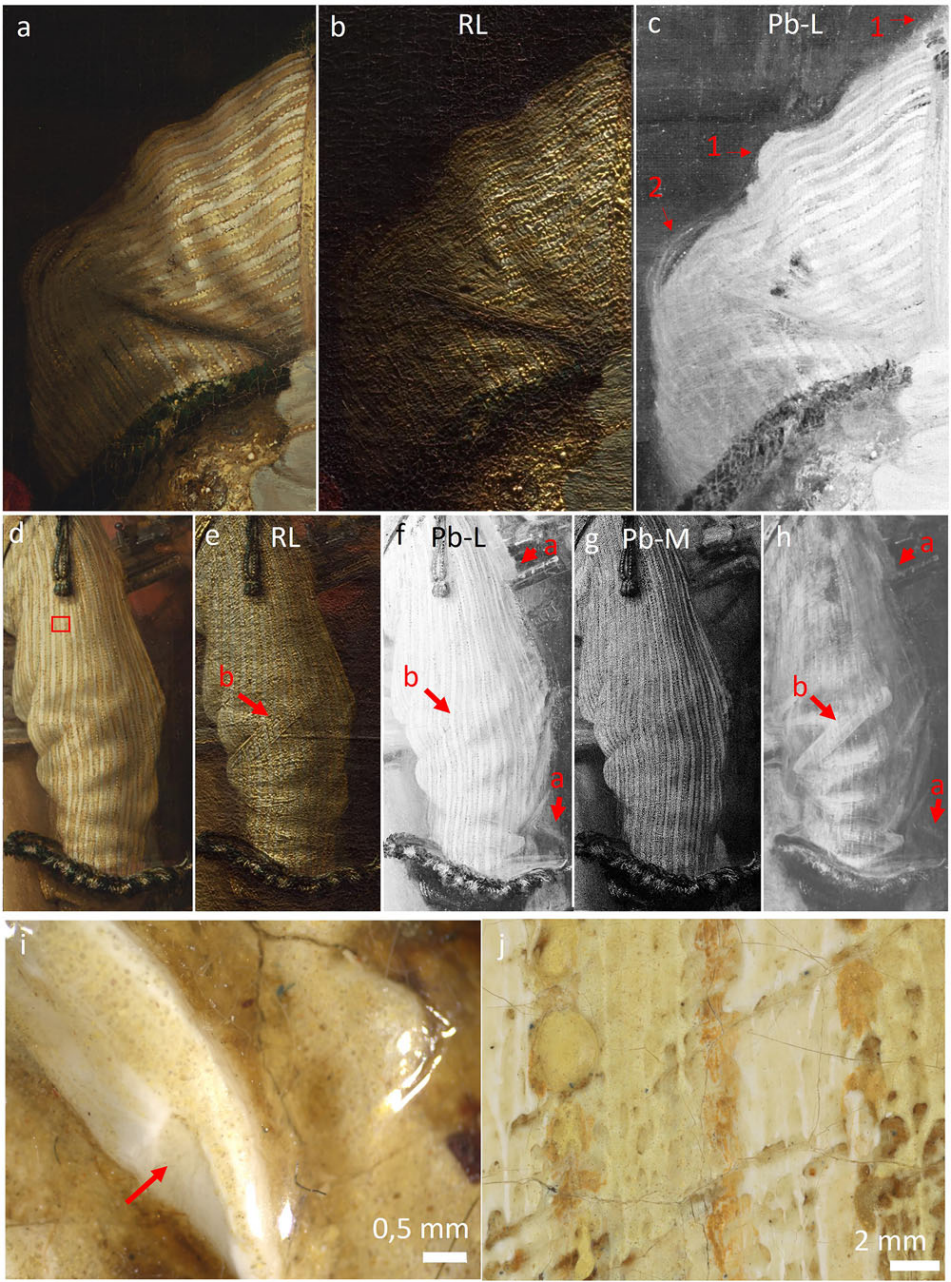
Textured lead white underpaint. Detail of Van Ruytenburch’s costume, proper right sleeve in (**a)** visible light, **b** raking light (illuminated from the top), **c** corresponding MA-XRF element distribution of lead (Pb-L), with red arrows 1 indicating the contour of the sleeve left in reserve and arrow 2 indicating the later contour adjustment of the sleeve. Detail of the proper left sleeve in **d** visible light, **e** raking light, **f** Pb-L distribution, **g** Pb-M distribution, **h** X-radiograph. Red arrow a, indicating the initial contour of the sleeve in the early steps of the paint process, red arrow (**b)**, indicating the textured lead white underpaint. **i** Stereomicroscopic detail of the lead white underpaint used to build-up the embroidery (red arrow points towards the exposed underpaint, the location and OCT-heightmap can be found in Fig. S1). **j** Detail of the 5 µm-high resolution photograph, location indicated by a red box in (**d**) showing the paint of the yellow and white stripes of the doublet skipping over the thick textured underlying lead white-containing underpaint.

Based on examination of the X-radiograph, Van de Wetering and those who investigated the painting in the 1970s concluded that Rembrandt painted *The Night Watch* from the back to front, starting with the background and ending with the figures in the foreground^2^. In doing so, Rembrandt typically left a “reserve” for the foreground figures when laying in the background. Based on the lead MA-XRF distribution map (Pb-L), we see that the silhouette of Van Ruytenburch was left in reserve (Fig. 2c, arrows 1) and that during the painting process, the contours of the sleeve and shoulder were adjusted with paint layers that overlap the background paint (Fig. 2c, arrow 2), a feature typically observed in Rembrandt paintings.

Through observations under the stereomicroscope and in raking light, in correlation with the lead MA-XRF distribution map (Pb-L), Rembrandt seems to have built up Van Ruytenburch’s costume, in particular the textured surface relief of Van Ruytenburch’s embroidery with multiple lead white based underpaint layers. This had already been proposed by Van de Wetering in 1976, based on examination of the X-radiograph (Fig. 2h)^14^. The high relief impastos, ranging up to 850 µm in height based on OCT (Fig. S1) and 3D surface mapping with the digital Hirox microscope, are found in the illuminated areas of the costume, while smoother brushstrokes are present in the shadow areas. This corresponds with differences in intensity of the lead signal. MA-XRF provides elemental information about the paint surface, but does not yield depth-selective data. The distribution of the lead L-line shows information about both the lead white impastos at the surface and the lead species located deeper in the paint stratigraphy. The lower energetic lead M-line distribution, however, provides only information on the lead compounds present at the paint surface. By closely observing the paint surface in visible (Fig. 2d) and raking light (Fig. 2e) while considering the Pb-L (Fig. 2f) and Pb-M maps (Fig. 2g), we can discriminate lead containing brushstrokes that stem from the underpaint, and the initial contouring of the sleeve (Fig. 2f, h, red arrows a), and do not correlate with lead-containing species at the surface. An example is the underlying pronounced broad brushstroke in the proper left sleeve of Van Ruytenburch’s doublet. This stroke was swiftly applied to indicate the fall of the fabric, which is best visible in raking light and with the naked eye (Fig. 2e-d). This textured underlying brushstroke is placed where the light hits the fabric and functions as a guide for the fabric modeling and the direction of the white and yellow stripes in the subsequent paint layers (Fig. 2e, f, h, indicated by red arrows b). The thick lead white underpaint is also used to mimic, among others, the threads of the embroidery (Fig. 2i) and the rough-grained texture of the leather in the jerkin. The lead white underpaint, therefore, is used both for imitating material textures and for creating a spatial illusion. In some places, the cream-colored lead white underpaint is visible on the paint surface and seems to function as a base tone for scumbles in the shadowed areas. A scumble is an opaque paint layer that allows the color underneath to show through by being applied either very thinly or irregularly^52^. The pronounced textured lead white underpaint was fully dried before Rembrandt continued applying the subsequent paint layers. This is visible where the paint of the yellow and white stripes of the doublet skips over the thickly textured underlying brushstroke (Fig. 2j).

**Fig. 3:**
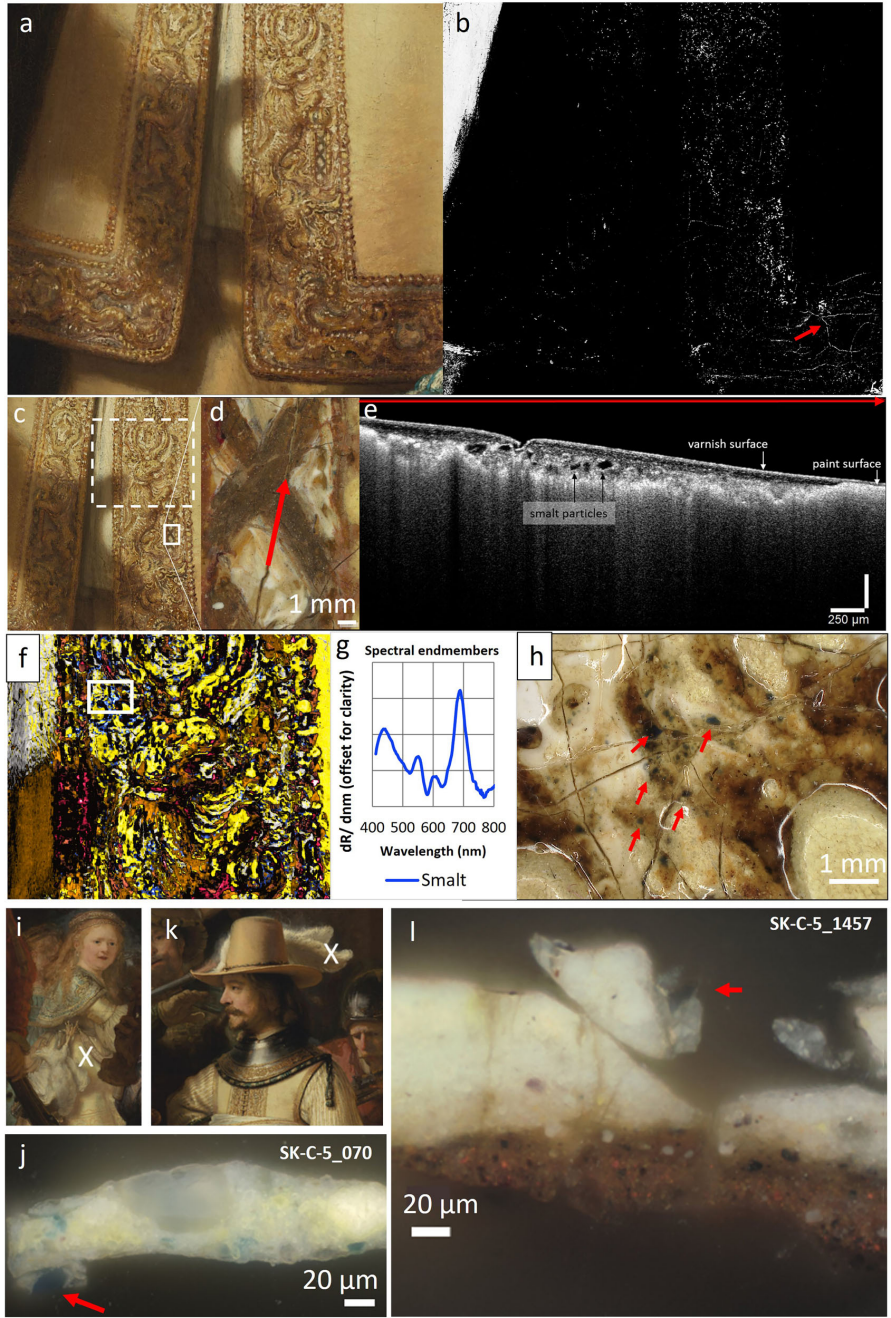
Smalt in the lead white underpaint. **a** Visible image of the embroidery and **b** corresponding cobalt MA-XRF distribution (Co-K). The red arrow in (**b**) indicates the cobalt signal, which follows a crack pattern in the embroidery. **c**, **d** details of the embroidery (**e**) OCT cross-sectional image obtained in the crack indicated by a red arrow in (**d**). **f** Detail of the composite SAM-map (Spectral Angle Mapper) discussed in Fig. 5, indicating the distribution of intact smalt (**g**) in dark blue. **h** Stereomicroscopic detail showing exposed underpaint with blue smalt particles (red arrows) as mapped by the SAM-map (white square in (**f**)). **i** Sample location and darkfield image (**j**) of cross-section SK-C-5-070 taken in the yellow dress of the girl, showing a smalt particle (red arrow) in a lead white containing underpaint. **k** Sample location and dark field image (**l**) of cross-section SK-C-5_1457 taken from Van Ruytenburch’s feather (smalt particle indicated by a red arrow).

**Fig. 4:**
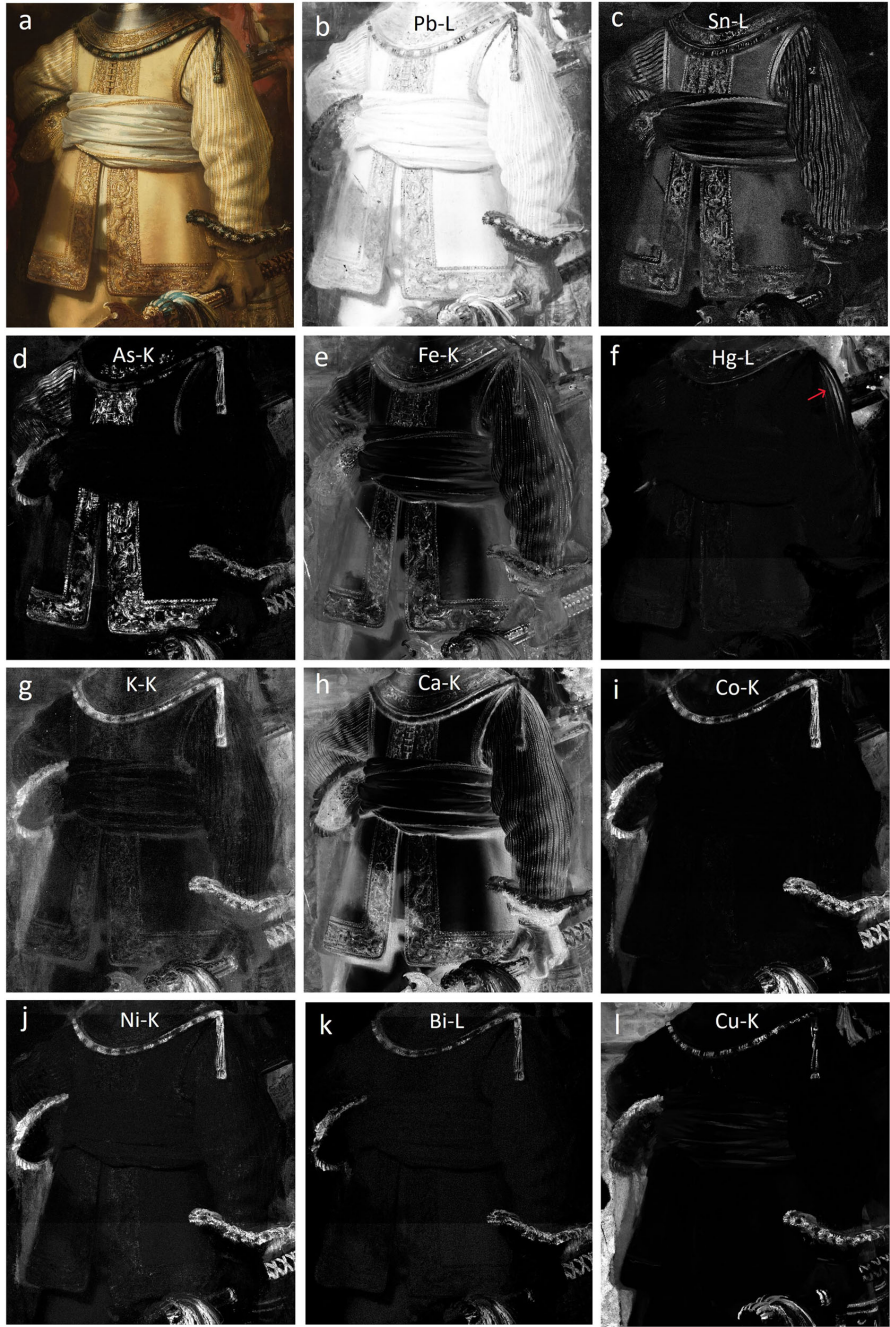
MA-XRF results. **a** Visible image and corresponding elemental distribution maps for **b** lead (Pb-L), **c** tin (Sn-L), **d** arsenic (As-K), **e** iron (Fe-K), **f** mercury (Hg-L), **g** potassium (K-K), **h** calcium (Ca-K), **i** cobalt (Co-K), **j** nickel (Ni-K), **k** bismuth (Bi-L), and **l** copper (Cu-K).

Close examination of the paint surface, OCT measurements, MA-XRF and RIS in the VNIR range revealed the presence of smalt in the lead white textured underpaint, a pigment that was not identified in *The Night Watch* in past material studies (Fig. 3). Smalt is a blue cobalt glass pigment made by melting a source of silica, potash as a flux and “zaffre”, a mixture of roasted cobalt ores and quartz or sand^22,53,54^ The blue pigment particles were observed under the stereomicroscope associated with the cream-colored underpaint in the buff jerkin and metal gorget. In particular, in the deeper paint cracks, clustering of blue particles could be observed. OCT measurements in a paint crack at the St-Andrews cross in the embroidery revealed the presence of angular particles of smalt (Fig. 3c-e). By stretching the intensity levels of the cobalt MA-XRF distribution map (Co-K), the presence of cobalt, correlated to nickel (Fig. 4j), can be demonstrated within the paint cracks and in between the impastos in the embroidered border (Fig. 3b–red arrow). With MA-XRF, the presence of smalt is indicated through the correlation of cobalt and nickel, potassium and in most cases arsenic^21,22,55^. Also, smaller concentrations of iron and bismuth can be found^56^. The cobalt signal in these areas was previously rendered invisible due to the presence of areas in the costume more abundant in smalt and could have been easily overlooked. With RIS-VNIR, we were able to confirm and map the still intact blue smalt particles in between the impastos and in the cracks, in locations where they were not covered by subsequent paint layers (Fig. 3f highlighted by white square and 3 h–red arrows).

A cross-section from the girl in *The Night Watch* also reveals a lead white underpaint containing particles of smalt that is used for the build-up of the lead-tin yellow containing dress (Fig. 3i-j). In a cross-section taken from the white feather of Van Ruytenburch’s hat (Fig. 3k-l) a single smalt particle is also found in the lead white underpaint. The addition of smalt in the underpaint layers or to obtain textured paint is not a new observation and was already encountered in textured underlayers in Rembrandt’s *Susanna* and *The Standard Bearer* (both 1636) and *Homer* 1663^15,22,57,58^. In this way, Rembrandt manipulated the properties of the lead white paint to achieve a textured paint with a higher yield stress, which allows the structure of the brushstroke to be better preserved. As proposed by Van Loon et al., coarse smalt could have been added as a bulking agent to create volume and texture or for siccative purposes, as cobalt-containing pigments are known to accelerate the drying of oil^22,59^. The addition of smalt to the lead white underpainting as a catalyst in the drying of the oil and/or for rheological purposes had also been taken into account by Groen in 1997. Then, however, only a single particle of smalt was found in the lead white underpaint of Van Ruytenburch’s boot^16^. Now, with macroscopic elemental and molecular mapping techniques, we are able to obtain a more complete visualization of the use of smalt in the underpaint, where previous cross-section analysis could not reveal its full extent. It is possible that smalt was added both as a bulking agent as well as to provide a cooler hue to the underpainting for contrast with the subsequently applied lead white and lead-tin yellow impastos of the upper paint layers. Groen also reported a weight percentage of 5–10% (25% vol percentage) of chalk in the lead white underlayer in Van Ruytenburch’s white feather, detected with XRD, which was hypothesized to have been added to manipulate the rheological properties of the paint to obtain a more “viscous” paint^15,60^. Re-examination of the same cross-section with SR-µ-XRPD confirmed the presence of calcite; however, only a few tiny particles were detected, which does not approximate the 25% vol suggested by Groen.

Groen also studied the addition of proteinaceous material for rheological purposes in lead white impasto paints in Rembrandt paintings. Aside from linseed oil, she detected amino acids from a proteinaceous material in a sample from Van Ruytenburch with HPLC and tentatively identified the source as egg. To confirm this, and more specifically identify the type of proteins in areas of impasto in Van Ruytenburch’s costume, two new samples were taken from the border of the jerkin, at the height of his armpit, near an abraded area, from a lead white impasto (SK-C-5_089) and a lead-tin yellow impasto (SK-C-5_090) (Fig. S2). The samples were analyzed with nano LC-MS, but no proteinaceous material was detected in the new samples. This could mean that there is no protein or only a very low amount is present in these particular samples, or that the proteins are too heavily cross-linked with the linseed oil and could not be extracted. Lluveras-Tenorio et al. studied the chemistry of protein-drying oil mixtures and how oil curing in the presence of proteins affects protein analysis using mass spectrometric techniques. They emphasize the challenges of protein analysis in cultural heritage objects and highlight how the copolymerization of lipids and proteins, leading to highly cross-linked and insoluble polymers, can impact protein detection^61^. More recently, research by Dietemann et al.^62^ and Van Ewijk at Akzonobel^63^ showed that impasto paint could be obtained via other means: a small droplet of egg(yolk) or a little bit of water added to lead white oil paint already suffice to substantially change the rheology or material properties of a paint through structures called capillary suspensions; the paint becomes very stiff due to the high surface tension of water^62^.

A recent study by Gonzalez et al. identified the presence of plumbonacrite (Pb_5_(CO_3_)_3_O(OH)_2_) in selected samples of Rembrandt’s lead white impasto paints, including samples from *The Night Watch*, taken from Van Ruytenburch’s feathers and the collar of Rombout Kemp, the figure at the far right. The authors hypothesize that this degradation product (not an original paint component), which forms under alkaline conditions, indicates the use of a lead-based drier in the oil medium, possibly due to the use of litharge-treated oil, to also obtain lead white impastos^64^. We conclude from the above that Rembrandt used various means to achieve a textured underpainting, as it is an instrumental step in effectively planning and executing his light-shadow modeling and depiction of material surface textures.

### The embroidered buff jerkin and doublet: *pigment use*

**Fig. 5:**
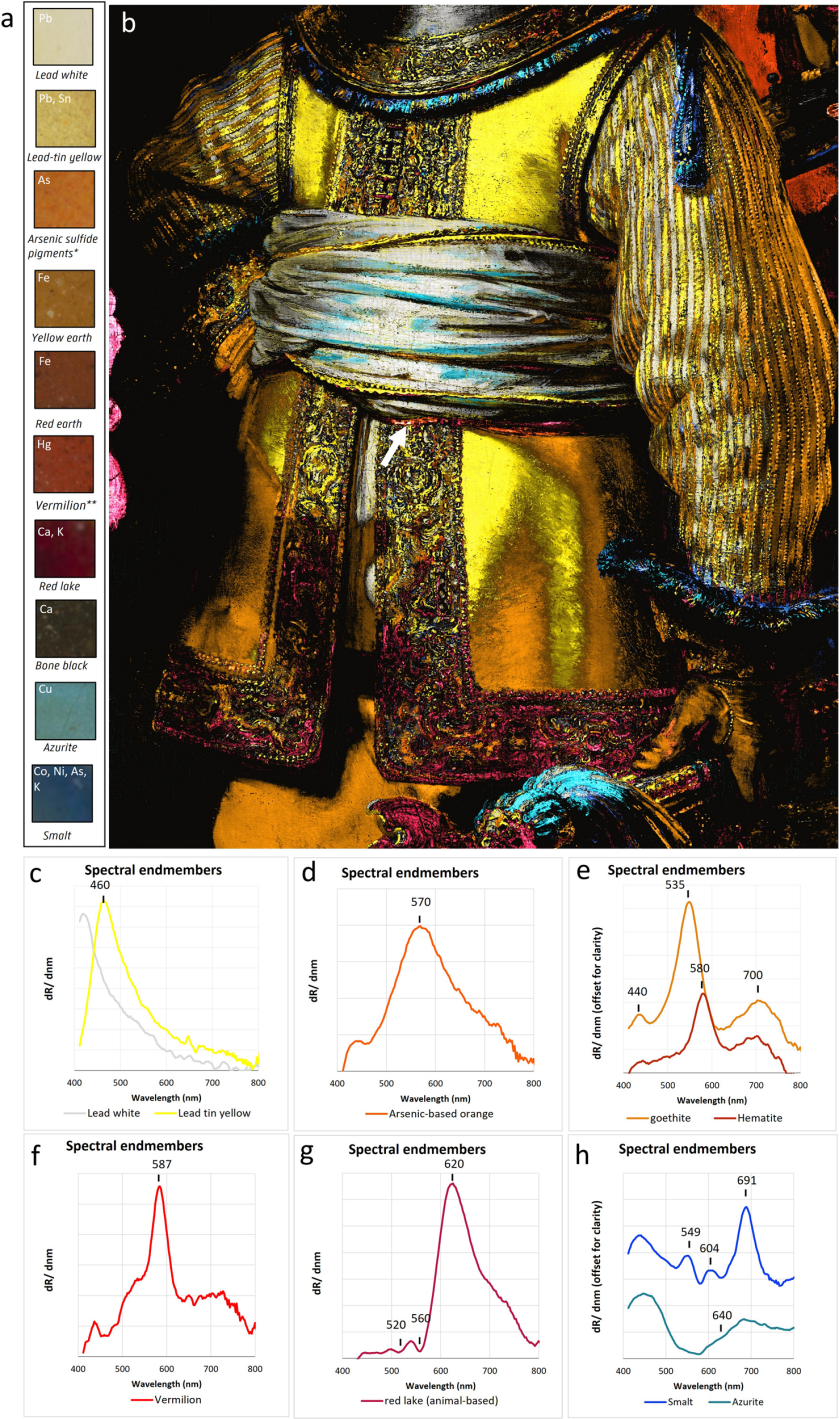
RIS-VNIR results. **a** Detailed squares of the 5 µm resolution photograph which were selected to generate the first derivative reflectance spectral endmembers used to obtain SAM-maps. **b** Composite overlay of the colored SAM-maps showing **c** lead white (white-gray) and lead-tin yellow (yellow), **d** an arsenic-based orange pigment (orange), **e** goethite (light-brown) and hematite (red brown), **f** vermilion (red), **g** an animal-based red lake (pink), **h** azurite (cyan) and smalt (dark blue).

The MA-XRF maps of the most relevant elements for pigment identification are shown in Fig. 4. The RIS-VNIR results are shown in Fig. 5 which displays a composite overlay of the maps generated through the SAM algorithm, using the 1st derivative reflectance spectral endmembers for lead white (white), lead-tin yellow (yellow), goethite (light brown), hematite (red brown), an arsenic-based orange (orange), smalt (dark blue), vermilion (red), azurite (cyan), and an animal-based red lake (pink). The separate gray-scale SAM-maps can be found in Fig. S3. The endmembers were manually selected based on the elemental information and in close observation with the paint surface to obtain the most accurate distribution of the individual pigments (Fig. 5a). The SAM-maps only show where the pigments are used in nearly pure form; areas containing heavily mixed pigments are not shown, thus providing a more clear visualization of the pigments used in the illuminated and shadowed parts.

### Lead white–lead-tin yellow

Lead white and lead-tin yellow were abundantly used in the costume, specifically in the illuminated areas. They correlate to thick, bright impasto brushstrokes that were applied over the already textured underpaint to render three-dimensional aspects of details in the costume, such as the borders of the buff jerkin. For the highest highlights, pure lead white strokes were applied on top of the lead-tin yellow impasto. Lead white, composed of a mixture of lead carbonates (discussed more in depth later), is visible in the lead MA-XRF distribution map (Pb-M (Fig. 2g) and Pb-L (Fig. 4b)) and lead-tin yellow is identified through the co-presence of lead and tin (Pb-L and Sn-L (Fig. 4c)). In the 17th century, two types of lead-tin yellow were available. Lead-tin yellow type I, Pb_2_SnO_4_, is the more frequently used, while type II (PbSn_1-*x*_Si_*x*_O_3)_ contains additional silicon (Si)^65,66^. Silicon was mapped with MA-XRF, however, in *The Night Watch* it predominantly correlates with the presence of smalt (a silica glass). The apparent absence (according to MA-XRF) of silicon in the areas rich in lead-tin yellow should be interpreted carefully as MA-XRF is less sensitive for the detection of (low energy) X-rays emitted from low Z elements such as silicon. RIS-VNIR permitted us to identify lead-tin yellow type I, the lead ortho-stannate phase, based on the typical spectral features (a maximum peak in the first derivative at 460 nm; Fig. 5c)^12,67^. The identification of type I lead-tin yellow was also confirmed through SEM-EDX analysis of several cross-sections and by SR- and MA-XRPD mapping, discussed below.

### Earth pigments

Also, earth pigments, visualized by the Fe-K MA-XRF map (Fig. 4e), were identified in Van Ruytenburch’s costume. RIS-VNIR enabled us to differentiate between the presence of yellow and red earth pigments by mapping the spectral features of goethite (α-FeO(OH)) and hematite (α-Fe_2_O_3_), which are indicative, respectively, of the pigments yellow earth and red earth (Fig. 5b,e). Goethite has a maximum peak in the derivative spectrum around 535 nm accompanied with a small peak at 440 nm, and a broad peak around ~700 nm, while hematite typically has a maximum peak in the derivative spectrum at 580 nm and a broader peak at ~700 nm^12,68^. This shows that Rembrandt only sparingly used red earth in Van Ruytenburch’s costume; it is only found in the shadow cast by the white sash around his waist (Fig. 5b, white arrow), while yellow earth can be found in the majority of the shadowed parts of the costume.

### Arsenic-based pigment

MA-XRF revealed the presence of an arsenic-based pigment, which is discussed in depth below. For the RIS-VNIR composite, a 1st derivative spectral endmember was taken from an orange highlight following the MA-XRF distribution map and stereomicroscope observation (Fig. 5a, As). While the asymmetric peak at 570 nm (Fig. 5d) suggests the presence of the orange-red realgar^12,67^, an in-depth study showed the unusual presence of a mixture of pararealgar and amorphous pararealgar^69^. The SAM-map shows a limited distribution compared to the MA-XRF As-distribution, as it is present in underlying layers and due to degradation (see “Discussion” below).

### Vermilion

The bright red mercuric sulfide pigment vermilion (HgS), visualized by the Hg-L MA-XRF distribution map in Fig. 4f (red arrow), is only scarcely present in the costume. Rembrandt applied this red pigment in three lines on the white and yellow striped doublet at the proper left shoulder on the shadow side. Rembrandt must have applied it as a reflection of the red costume of the musketeer loading a gun, depicted standing closely to his proper left. *Weerglans*, or reflection of the neighboring surfaces, are pictorial clues employed by many 17th-century artists to render depth and imitate (reflective) material surfaces. It tells us something about the reflective properties of the yellow and white striped doublet. A weak signal of mercury is also visible in the embroidery. A cross-sectioned sample taken for the identification of the arsenic sulfides (SK-C-5_017) confirmed the presence of a small amount of vermilion mixed with lead-tin yellow for the orange paint in the underlying layers^69^.

### Lake pigments

RIS-VNIR allowed the mapping of a red lake pigment based on the spectral features showing a broad and asymmetric peak in the derivative spectra with a maximum around 620 nm. The red lake pigment is animal-based (such as kermes, cochineal and lac insects) as typical absorption spectral features are visible at 520 and 560 nm (Fig. 5g)^70,71^. While RIS-VNIR provided insight into the origin of the dyestuff, MA-XRF offered insights into the substrate of the lake pigments. Both the calcium (Fig. 4h) and potassium MA-XRF map (Fig. 4g) correlate to the presence of the red lakes, following the SAM-map (Fig. 5b, S3) in close observation with the paint surface. Lake pigments are an important part of Rembrandt’s palette and are used to give depth and richness to shadows or create richly colored translucent glazes. From previous research, it is known that Rembrandt used various types of lake pigments either on their own or in complex mixtures with, e.g., earth pigments and bone black to create warm translucent browns for the shadows, or even mixed several types of lakes to obtain a greater range of translucent reds and yellows^31,59,72^. Typical red dyes, in Rembrandt’s time, were extracted from the madder plant [*Rubia tinctorium* *L*.], brazilwood and scale-insects such as cochineal and kermes, while common sources for yellow dyes were buckthorn berries, weld and dyer’s broom. For Van Ruytenburch, we could only produce convincing spectral endmembers for one type of lake pigment, while cross-section analysis suggests also the presence of a yellow lake, however, yellow lakes are prone to fading and are therefore difficult to identify in a noninvasive manner. This will be discussed in more detail in future studies. The SAM map for the animal based red lake also only partially maps the distribution of the red lake if we compare it in detail with the paint surface. This can be explained in various ways: pigment degradation, color fading and the presence of the red lake in mixtures with darker pigments such as bone black. This is consistent with the observation that the unmapped lake is found specifically in the deeper shadows of the costume.

### Bone or ivory black

As black corresponds to the total absorption of visible light, it is impossible to differentiate with RIS among different blacks, such as lampblack, vine black and bone black. These pigments show identical reflectance spectra with nearly 0% reflectance^67^. They are consequently not included in the RIS-VNIR composite. The rather black areas in the costume, however, could be correlated with the calcium MA-XRF map (Fig. 4h) in a similar manner to the red lakes. A phosphorus MA-XRF map was also obtained (not shown) and could be correlated with more intense calcium hotspots in the costume, suggesting the presence of bone black or ivory black pigments. As mentioned above, the limited detection power of MA-XRF for low-energy X-rays emitted from low-Z elements should be taken into account. The correlated detection of calcium and phosphorus was confirmed with SEM-EDX in paint cross-section SK-C-5_016a and with MA-XRPD through the identification of hydroxylapatite (Ca_5_(PO_4_)_3_(OH)) and whitlockite Ca_9_Mg(PO_4_)_6_(PO_3_OH) in the metal gorget (not shown). Black pigments are found in the gray and black tones used in the metal gorget and the three St. Andrew’s crosses in the embroidered border of his buff jerkin; they are also mixed with red lake pigments to render the deeper shadows in the costume.

### Blue pigments—azurite and smalt

Azurite could be visualized in the copper MA-XRF map (Fig. 4l, Cu-K) and also identified using RIS, based on a spectral endmember showing a slow rise in reflectance after 640 nm (Fig. 5h), typical features for the copper-based carbonate azurite (Cu_3_(CO_3_)_2_(OH)_2_). Azurite was also confirmed based on the identification of two spectral features at 2285 nm and 2352 nm using shortwave infrared (SWIR) spectroscopy^12^. These features correspond to the combination band of carbonate stretching and hydroxyl bending (δOH + νCO3^2−^) and the third overtone of carbonate stretching (3νCO3^2−^)^12,73^. The pigment corresponds to the lighter blue details in the embroidery and blue thick paint layers that were used in the illuminated parts of the blue and white tassel, fur-edged cuffs and the blue and yellow fringes around the metal gorget. Some azurite mixed with lead white can also be found in the white sash. Rembrandt specifically used azurite to paint the half shadows where the light shines on the sash.

As mentioned in the previous section, also smalt was identified in the costume and is found where the lead white-based underpaint is exposed to the surface and in the deeper blue shadows of the blue paint layers. The distribution is visualized in the MA-XRF maps of cobalt (Fig. 4i), nickel (Fig. 4j), arsenic (Fig. 4d), and potassium (Fig. 4g). The SAM map was obtained with a spectral endmember showing the characteristic features bands of smalt that become transformed into three maxima in the first derivative spectra at 549, 604, and 691 nm (Fig. 5h)^12,67^.

### Use of arsenic pigment for the material imitation of gold threads

Based on the MA-XRF scans, we were able to demonstrate the presence of arsenic sulfide pigments in *The Night Watch*. Most of the arsenic (As-K) present in *The Night Watch* can be correlated with cobalt (Co-K), nickel (Ni-K) and bismuth (Bi-L), indicative of the presence of smalt, with the exception of the arsenic signal present in the embroidered border and costume of Willem van Ruytenburch. Different types and qualities of smalt were available in the 17th century and depending on the manufacture and treatment of the original cobalt ores (smaltite [Co, Ni]As_3-2_, erythrite [Co, Ni]_3_[AsO_4_]_2_.H2O and cobaltite ((Co, Fe)AsS)) arsenic was (partly) removed (e.g., by roasting it for purification)^74^. In *The Night Watch*, however, and in other paintings by Rembrandt, arsenic is still (partly) present^21,22,72,75,76^.

**Fig. 6:**
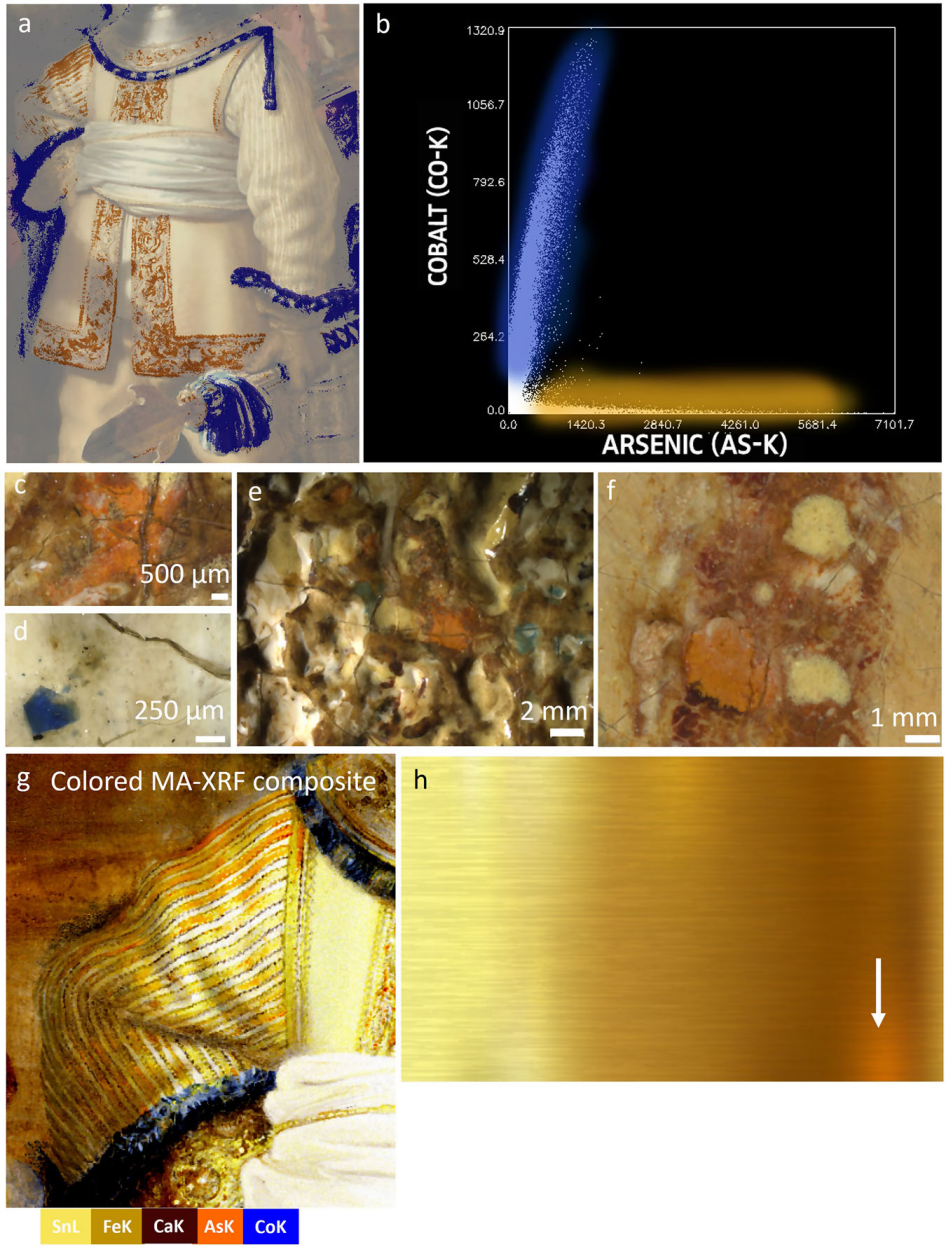
Co:As correlation biplot. **a** False color map (overlaid for visibility on the visible image) of the As-K: Co-K intensity biplot (**b**) showing two distinct groups. Pixels colored blue: As correlated to Co; pixels colored orange: As uncorrelated to Co. **c**, **e**, **f** Stereomicroscopic images of the orange arsenic-containing paint (**d**) microscopic image (Hirox) of an intact blue smalt particle. **g** Composite of inverted and colored MA-XRF elemental maps for tin (Sn-L, yellow), arsenic (As-K, orange), iron (Fe-K, light brown), calcium (Ca-K, dark red-brown) and cobalt (Co-K, blue). **h** image showing an example of a gold metal surface (orange glow reflection indicated with a white arrow).

To distinguish the arsenic stemming from smalt and the arsenic coming from arsenic sulfide pigments, a Co:As correlation biplot was used (Fig. 6a, b). For Van Ruytenburch’s costume, two distinct groups of pixels can be observed. One group shows a correlation between Co and As and can be linked to the pigment smalt (Fig. 6d), shown in blue. This correlates to the blue paint layers used for the partisan and decoration of the gorget. The second group shows As that is uncorrelated with Co and corresponds to the As_x_S_y_ pigments, shown in orange. The arsenic pigments are specifically found in the embroidered border and in Van Ruytenburch’s proper right sleeve. These areas can be traced back to a bright orange paint (Fig. 6c, e, f). In the embroidery it is mostly present as an underlying paint layer and only in some areas present at the surface (either due to abrasion or intentionally) such as the highlights in the shadowed areas (Fig. 6f). In the sleeves, arsenic is present at the surface and correlates to the warm golden stripes of the white and yellow striped doublet. This was confirmed with paint cross-section SK-C-5_016, taken from this area. Due to degradation (discussed below), the paint layers have a more brittle and broken-up gray-whitish appearance.

To study the function of the bright orange paint, an overlaid composite was made with a selection of the elemental MA-XRF distribution maps (Fig. 6g) (the separate selected elemental MA-XRF distribution maps are shown in Fig. S4). The maps were inverted and colored in Photoshop (the colors for the maps were chosen following the RGB values of the high-resolution image with the sampling tool). The aim of the composite image is not to approximate the original intention of the artist for reconstruction purposes but to serve only as a simple overlay to better study the purpose of the arsenic-containing orange paint. In this way, we see that Rembrandt employed the orange paint to obtain a warm orange, golden glow reflection, in imitation of the gold threads. The warm orange, golden reflection of gold in some light conditions is due to its unique optical properties/the way it interacts with light. Gold is a metallic element that has a characteristic yellow color, but its reflection of light can vary depending on the surface finish and the angle of incident light. In some light conditions, such as when it is illuminated by warm, low-angle sunlight with a yellow-orange tint, gold appears to have a warm, orange-golden reflection (Fig. 6h, white arrow). This is because a higher proportion of longer-wavelength red and orange light emitted by the light source gets selectively reflected by the gold, while shorter wavelengths are absorbed^77,78^. This selective reflection of warm colors can enhance the richness and warmth of gold’s appearance, creating the beautiful warm orange golden reflection that we often associate with this precious metal.

Rembrandt and contemporaries undoubtedly studied these optical phenomena and tried to imitate them, as exemplified here in Van Ruytenburch’s costume. The arsenic sulfide pigments, such as yellow orpiment [from Latin *auripigmentum*, golden pigment] (As_2_S_3_) and orange-red realgar (As_4_S_4_), are often suggested in contemporary sources for the pictorial imitation of gold^37,79^. In the contemporary artist’s manual *The Big World Painted Small*, Willem Beurs (1692) recommends painting gold with realgar, burnt umber in the shadows, sometimes mixed with a bit of lake, and highlights painted with King’s yellow (the synthetic equivalent of the mineral orpiment) or lead-tin yellow^37^. The pigment palette recommended by Beurs closely resembles the palette used by Rembrandt, with the exception for umber, where he used bone black, and he did not use King’s yellow for the highlights but lead-tin yellow. Samuel van Hoogstraten mentions that, next to yellow pigments such as yellow-ocher, lead-tin yellow and yellow lake, in particular orpiment might be used to render expensive clothes^3^.

The presence of the arsenic sulfides in Van Ruytenburch’s costume, specifically to imitate the optical properties of the gold threads, underlines the mastery of 17th-century painters in their search for a realistic rendering of material surface textures. And although several handbooks and guides were available with detailed instructions on how to paint different objects such as Willem Beurs’ manuscript, painters such as Rembrandt must have also made observations from real life. In the inventory of the sitter Frederick Rihel, for instance, various clothing and weaponry were listed, including a gold-embroidered buff leather coat, in which Rembrandt depicted him in his equestrian portrait, *Portrait of Frederick Rihel on Horseback* (probably 1663)^80,81^. Among the items in Rembrandt’s inventory, which included a large collection of arms and armor, a quantity of ancient textiles of diverse colors were found in the small painting room [“*een pertije antieckse lappen van diversche coleuren”*, ‘*Op de Cleijne Schildercaemer, in ‘t vijfde Vack’*], which he probably used to study their patterns, colors and material properties^82^.

### The organization of tonal steps—light to dark

**Fig. 7:**
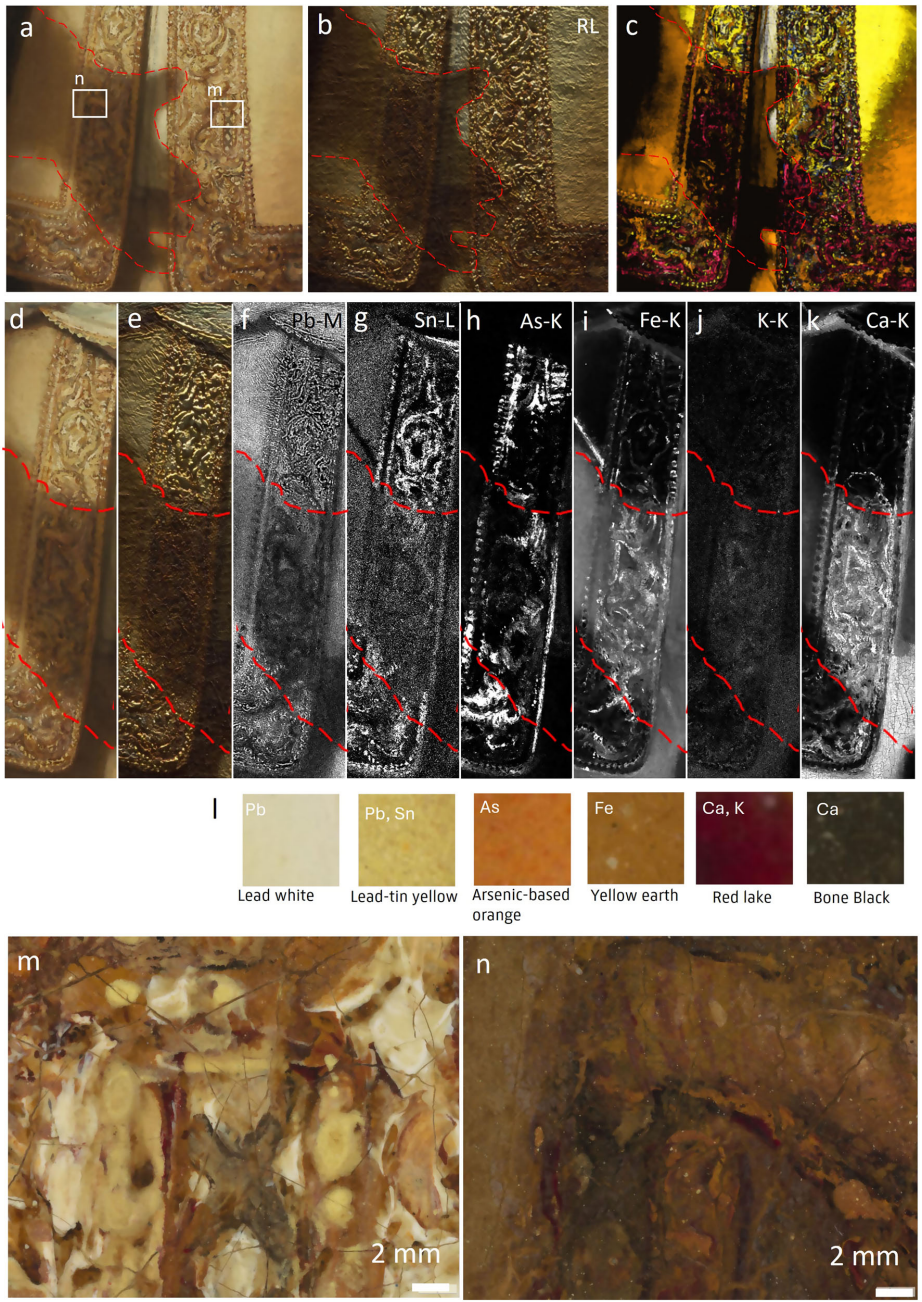
(Tonal grouping) Difference in build-up and pigment use for the cast shadow of Bannick Cocq’s hand on the embroidered buff jerkin of Van Ruytenburch and the illuminated side. **a** Visible light image with details (m, n) indicated with a white square and the outline of the shadow of the hand traced by a red striped line, (**b)** raking light image, **c** detail of composite image of SAM-maps (Fig. 5b), detail of the embroidered border in (**d)** visible light, **e** raking light and corresponding MA-XRF maps: **f** lead (Pb-L), **g** tin (Sn-L), **h** arsenic (As-K), **i** iron (Fe-K), **j** potassium (K-K) and **k** calcium (Ca-K). **l** pigment palette that was used for the tonal grouping. Details from the 5 µm resolution photograph of the Saint-Andrews crosses on the illuminated side of the embroidered border (**m**) and the shadow side (**n**).

The composite overlay of the SAM-maps (Fig. 5b) shows Rembrandt’s ability to manipulate colors and how he consistently chose specific pigment groups to portray the light and shadow tones and tonal variations in between. From the most illuminated parts to the deepest shadows, a systematic application of pigments and careful layering can be observed, contributing to the creation of a convincing *chiaroscuro*, ultimately achieving compositional unity. A clear example is the embroidered border, which features the cast shadow of Banninck Cocq’s outstretched hand on Van Ruytenburch’s costume (Fig. 7). Rather than simply painting the shadow on top of the embroidery, Rembrandt meticulously planned its placement from the initial stages of the work.

In the illuminated parts of the embroidery, Rembrandt adheres to specific rules: sharp tonal contrasts, more vibrant hues and an extended pigment palette, using predominantly lead white (Fig. 7f), lead-tin yellow (Fig. 7g), yellow earth (Fig. 7i), arsenic-based orange for the golden glow (Fig. 7h); in the cast shadow, on the other hand, red lakes and bone black (Fig. 7 j-k) are used. This is applied on top of the 3D textured underpaint, which was done in the earlier paint stages (visible in raking light, Fig. 7b, e). Rembrandt builds up consecutively from the shadowed tones: yellow earths, red lake with sometimes a bit of bone black, to the top layers with thick lead-tin yellow and lead white impastos.

In the deeper shadows, Rembrandt uses more subtle color transitions, less details and smooth paint layers such as glazes and scumbles rather than impastos (Fig. 7b, e). The pigment palette consists mainly of yellow earth pigments as a base, as seen for the casted shadow of the hand in the iron map, red lake glazes, and bone black scumbles to indicate the design and the arsenic-based orange for a golden undertone or reflection (Fig. 7i-k). Depending on the light conditions, the brightest highlights are realized with yellow earth or with a scumble containing lead-tin yellow.

Rembrandt's principle of grouping pigments according to the appropriate lighting conditions and his systematic organization of tonal pigment steps align with Samuel van Hoogstraten’s writings on Rembrandt’s painting technique: to not overly blend lights and shadows, but rather to skillfully combine them in groups. He emphasizes the power of gentle accompaniment, suggesting that when strong lights are accompanied by lesser lights, their radiance is enhanced. Similarly, he highlights the importance of surrounding deepest darks with lighter darks to intensify the impact of light^3^. The pigment chosen for the brightest highlight is always dependent on the grade of illumination. An example is given of the treatment of Van Ruytenburch’s pearled hat decoration in Fig. S5 following the grades of illumination as described by Samuel van Hoogstraten.

### Simultaneous contrast effect

The illusion of light and shadow to create depth and form is the most challenging aspect of 17th-century painters, and especially the half tone (and the transition) is crucial. According to observations of paintings by Bomford et al.^59^, Rembrandt used alternating warm and cool colors. Reflections into shadows are cool, the shadows themselves are warm; half-tones are cool, lights warm, and the brightest highlights are cool again.

**Fig. 8:**
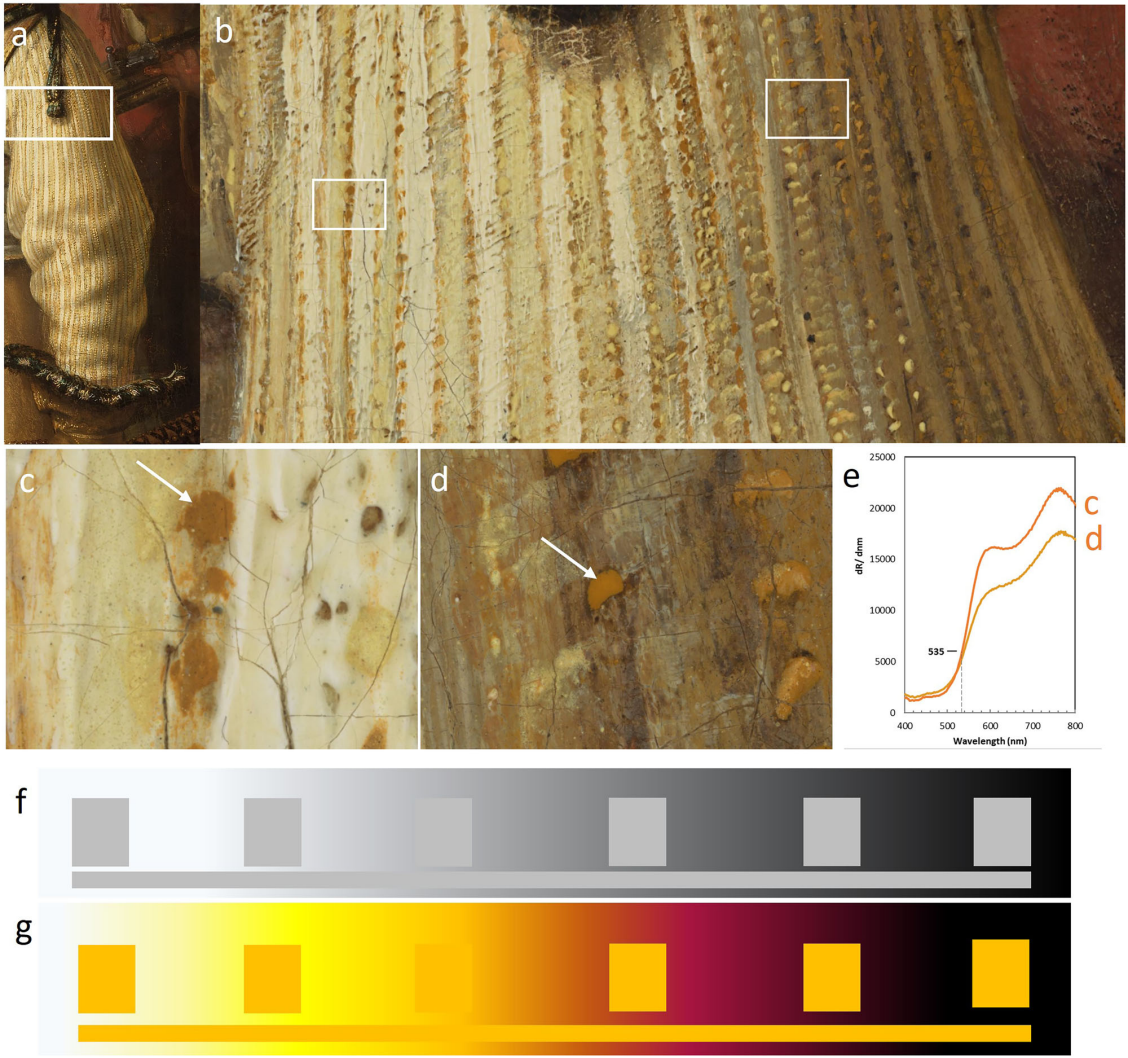
Detail of Van Ruytenburch’s doublet sleeve. **a**, **b** Visible light details of the 5 µm resolution photograph. The areas shown in (**c**) and (**d**) are both shown as white boxes in (**b**), respectively. **e** The reflectance spectra from the yellow ocher dots indicated with white arrows in (**c**, **d**). **f** Example of a brightness simultaneous contrast illusion (**g**) Example of a color simultaneous contrast illusion.

Rembrandt seems to have used a sophisticated approach to accommodate the light and shadow transition in Van Ruytenburch’s sleeve. A yellow ocher paint was used to imitate the stitches along the white and yellow striped doublet for both the illuminated and the shadow side (Fig. 8). On the illuminated side (Fig. 8c) the yellow ocher dot appears visually darker next to the lead white and lead-tin yellow impastos, while the yellow ocher dot on the shadow side (Fig. 8d) appears lighter and cooler as it is surrounded by more bone black and lake pigments. The extracted VNIR spectra from the yellow ocher dots, however, for both spectra show a reflectance transition edge at 535 nm (Fig. 8e), which means that they have the same color hue. There is a slight difference in the overall reflectance of the spectra, with c showing a lighter reflectance, although this dot is actually perceived as darker. The higher reflectance here is likely caused by the contribution of the lead white underlayer.

This particular effect reminds us of the simultaneous contrast effect, which is an optical illusion that exploits how the human visual system processes light and shadow and how our perception of these phenomena is shaped by the context in which they are perceived^26,83^. This is, for instance, demonstrated in Fig. 8f-g where the perceived brightness of a medium-gray area or color of a yellow patch changes depending on the brightness or color of the adjacent areas. A medium-gray square (Fig. 8f) will look darker against a white background and lighter against a black background. Again, the actual luminance of the gray square remains constant, but our perception of its brightness changes. In Fig. 8g the yellow color is perceived more brown against a bright background and cooler yellow against a dark background.

With RIS-VNIR, in essence, we can prove that rather than using different tonalities or paint mixtures, Rembrandt specifically employed one single yellow ocher-based paint to mimic actual optical phenomena. This effect not only makes the light areas appear more radiant and the dark areas deeper, achieving a stronger light-dark contrast that appears natural to the beholder, but it also enhances a sense of volume and three-dimensionality based on the application and placement of the yellow ocher dots following the shape of the folds. This shows that Rembrandt was not only acutely aware of ideas at the time regarding visual perception, but also applied them to render a convincing *chiaroscuro*. Although the simultaneous contrast effect is a term that would be formalized much later, Ibn al-Haytham^84^, a renowned 11th century scientist and polymath, and Leonardo Da Vinci (1452–1519)^85^ already described similar phenomena where colors appear differently depending on the surrounding colors and lighting conditions.

### Insights from MA-XRPD—impact of pigment degradation

**Fig. 9:**
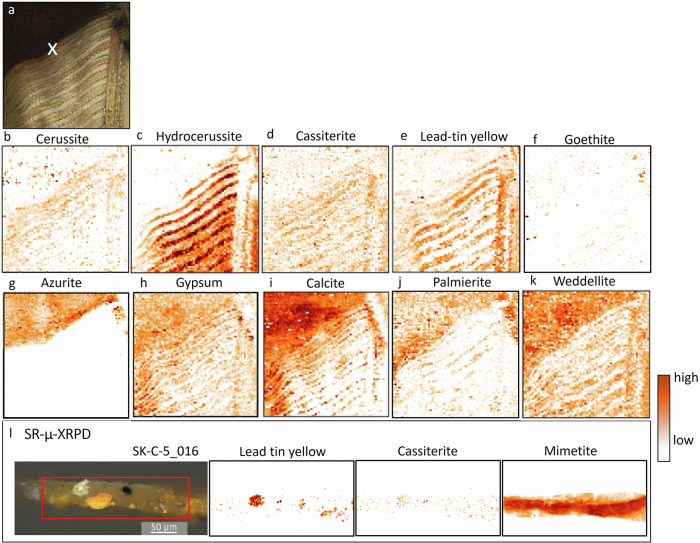
Results obtained with MA-XRPD at the proper right sleeve. **a** visible image with sample location SK-C-5_016 indicated with a white cross, distribution for (**b)** cerussite, **c** hydrocerussite, **d** cassiterite, **e** lead-tin yellow, **f** goethite, **g** azurite, **h** gypsum, **i** calcite, **j** palmierite, **k** weddellite. **l** A selection of the SR-µ-XRPD results on paint cross-section SK-C-5_016.

**Fig. 10:**
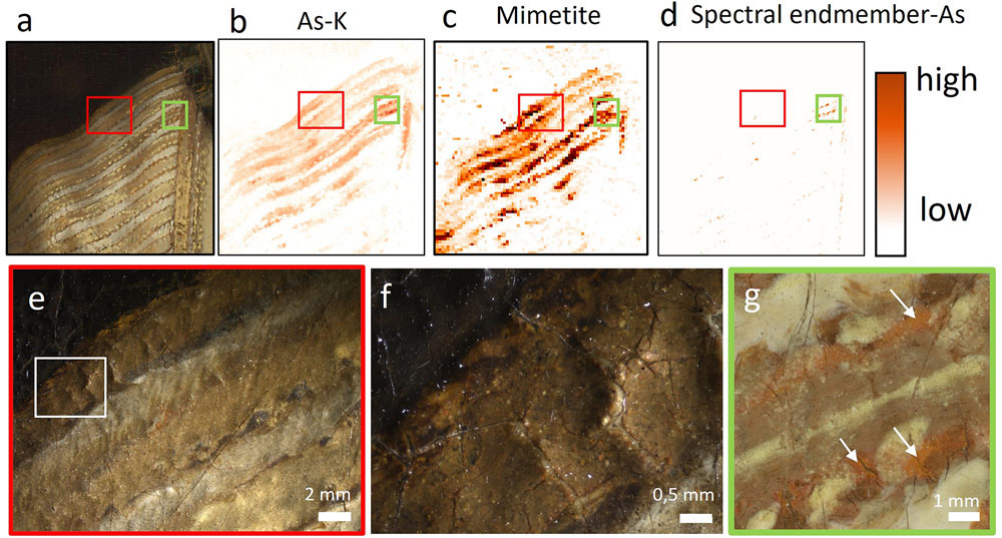
Degradation of the arsenic-based pigments in the proper right sleeve. **a** Visible image, **b** corresponding elemental MA-XRF distribution image of arsenic (As-K). MA-XRPD map for (**c**) mimetite. **d** SAM-map obtained with the spectral endmember showing characteristic features of an arsenic-based orange pigment, shown in Fig. 5d. Stereomicroscopic images at different magnifications (0.63× and 2×), showing the greyish haze caused by the formation of mimetite. Area (**e**) is indicated by a red square in (**a**-**d**). Area **f** is indicated with a white box in (**e**). **g** Detail of the 5 µm resolution photograph (location indicated by a green box (**a**-**d**)) indicating the orange arsenic containing paint (with white arrows) that are at the surface, not fully degraded and still detectable with RIS-VNIR (**d**).

**Fig. 11:**
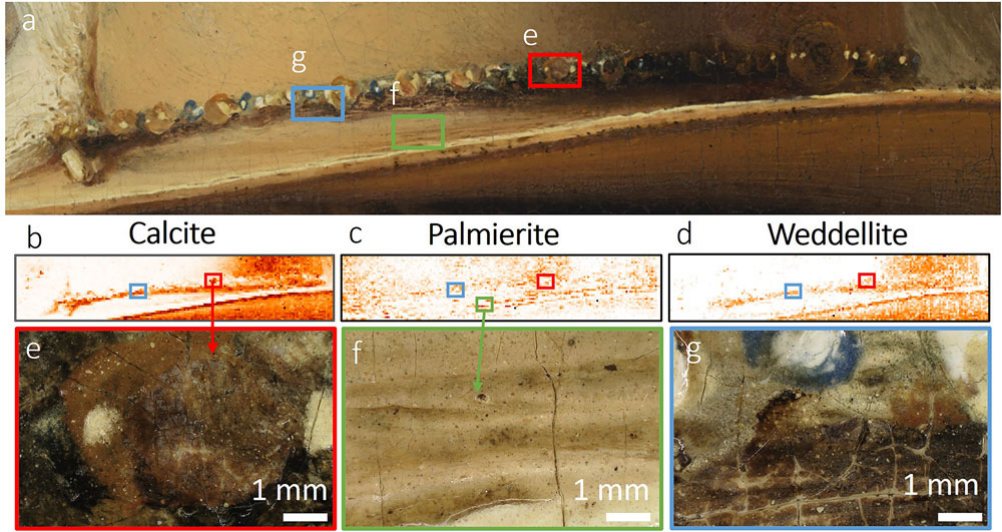
MA-XRPD maps of the pearls in Van Ruytenburch’s hat. **a** Visible image, (**b)** calcite, (**c)** palmierite and **d** weddellite. Digital microscopic images (Hirox) of (**e**) a pearl with a red lake shadow that has a whitened appearance and corresponds with the distribution of calcite (red box in (**b**)) and weddellite (red box in (**d**)). **f** the distribution of palmierite corresponds to the presence of smalt in the textured paint. **g** microscopic image of the whitened shadow under the pearled hat decoration corresponding to calcite (blue box in (**b**)) and weddellite (blue box in (**b**)).

In this section, a selection of the most relevant crystalline pigment phases and secondary degradation products will be discussed that were mapped at or close to the surface. Several areas were analyzed: the proper right sleeve, the embroidered border, the pearled hat decoration and the metal gorget^86^. MA-XRPD enabled us to confirm the pigments that were identified with RIS-VNIR: lead white (Fig. 9b-c), lead-tin yellow type I (Fig. 9e, Pb_2_SnO_4_), azurite (Fig. 9g, Cu_3_(CO_3_)_2_(OH)_2_), goethite (Fig. 9f, α-FeO(OH)), and additionally identified calcite (Fig. 9i, CaCO_3_) and gypsum (Fig. 9h, CaSO_4_·2H_2_O), probably associated with the substrate of lake pigments. The secondary degradation products found are mimetite (Fig. 10c, Pb_5_(AsO_4_)_3_Cl), palmierite (Fig. 9j, K_2_Pb(SO_4_)_2_) and weddellite (Fig. 9k, Fig. 11d, CaC_2_O_4_·2H_2_O).

Lead white is composed of a mixture of lead carbonates: cerussite (PbCO_3_) and hydrocerussite Pb_3_(CO_3_)_2_(OH)_2._ The mass ratio of those two compounds is influenced by many factors such as the production process (HC:C ratio differs for instance depending on the depth from metal to surface as proved by Gonzalez et al.^87^) and post synthesis treatments^88–90^, which may result in different lead white subtypes or qualities, each with different handling and optical properties^91^. MA-XRPD mapping (Fig. 9b, c) revealed a lead white with a high hydrocerussite (Pb_3_(CO_3_)_2_(OH)_2_ to cerussite (PbCO_3_) ratio. Regarding optical properties, although also highly dependent on the particle size^90^, a higher hydrocerussite to cerussite ratio is hypothesized to have a higher covering power and opacity^92^. These are optical properties which Rembrandt presumably chose to paint the brightest highlights, white passages and thick white impastos in the costume. Several secondary lead compounds such as lead(II) formate (Pb(HCOO)_2_), and plumbonacrite (Pb_5_(CO_3_)_3_O(OH)_2_) were further found to be present, and are discussed elsewhere^86^.

Aside from lead-tin yellow (Pb_2_SnO_4_), also an additional tin product was found: the lead oxide cassiterite (SnO_2_) (Fig. 9d). Cassiterite is a white to off-white pigment which according to De Mayerne, only a few painters such as Anthony van Dyck (1599–1641) experimented with in oil paint^36^. Its presence close to protrusions associated with lead saponification of the lead-tin yellow particles have been interpreted as a degradation product residue^93,94^. SR-µ-XRPD on cross-sections (SK-C-5_016), however, shows that the cassiterite particles are correlated with (intact) lead-tin yellow particles (Fig. 9l). Here it is therefore more likely to be the result of improper heating during the production process of lead-tin yellow^93–95^. Lead-tin yellow type I can be prepared by mixing lead monoxide (PbO), lead dioxide (PbO_2_) or lead tetroxide (Pb_3_O_4_) (or both components) with tin oxide (SnO_2_) and calcining them at elevated temperature. At higher temperatures, the decomposition of the final product can occur and thus the presence of PbO and SnO_2_ can be expected in the mixture^66,96,97^. Alternatively, tin oxide may have also been introduced after the cooling down process, to make the color of the yellow pigment intentionally lighter^98^. Rembrandt presumably opted for a more lemon-hued, i.e., lighter, lead-tin yellow pigment, as several varieties were available at the time, ranging from orange (calcined at lower temperatures) to bright lemon yellow (calcined at high temperatures)^98,99^.

The lead-tin yellow paint layers in Van Ruytenburch’s costume are overall affected by lead soap formation as the paint layer is characterized by a gritty (granular surface) texture commonly associated to the presence of small translucent white globules of lead soap aggregates.

Mimetite (Pb_5_(AsO_4_)_3_Cl) is a rare lead arsenate species that is considered to be a secondary degradation product that forms when soluble mobile arsenates precipitate out with available Pb^2+^ ions^30,100–104^. These mobile arsenate ions are present due to the degradation of the original arsenic sulfide pigments, pararealgar and semi-amorphous pararealgar^69^. Via MA-XRPD, mimetite (Fig. 10c) could be localized in the arsenic-rich regions of Van Ruytenburch’s costume (Fig. 10b) where the bright orange pigment was used at the paint surface to imitate the glow of the gold threads. From previous research, it is known that the transformation of arsenic pigments into secondary lead arsenates has a significant impact on the state and appearance of the paint layer, as the lead arsenates are colorless and transparent^30^. Paint cross-section SK-C-5_016 analyzed with SR-µ-XRPD, taken in the outermost border of the shoulder in the mimetite-rich area, also revealed that mimetite is distributed throughout the entire paint layer depth (Fig. 9l)^69^. From observations of the paint surface, the proper right sleeve of the costume is significantly affected as the paint layer is broken up and has a greyish transparent cloudy appearance with sporadically still intact yellow and orange areas where red arsenic particles are visible (Fig. 10e-f). RIS-VNIR did not yield convincing endmembers in these areas, likely due to the presence of the secondary degradation products, as this technique relies on the electronic transitions of pigments based on their color, and only maps areas where the original pigment is still intact. The paint layers with a higher concentration of the arsenic-based pigment mixture, and where it is present as an underpaint, are better preserved (Fig. 10d, g—intact areas indicated with white arrows).

Palmierite (K_2_Pb(SO_4_)_2_) has also been identified in various areas of the costume and the entire painting. Its formation also requires the presence and migration of Pb^2+^ ions. The source of these ions could be lead-based pigments, lead driers added to the oil or the lead-containing impregnation layer of the original canvas^86,105^. The K^+^ ions could derive from discolored smalt (due to the depletion of potassium from the glass), lake substrate (potassium alum) or from earth pigments (in which potassium is present as a minor element). Sulfur can originate from an internal or external source: internal as SO_4_^2–^ ions (potassium alum lake substrate) and external from environmental SO_2_^44,104,106–108^. This alteration product has been previously detected in paintings by Vermeer, Rembrandt and Jan Davidsz. De Heem and its formation is often co-localized with potassium-rich pigment sources such as red lake pigments and smalt^18,44,106,109^. In Van Ruytenburch’s costume, it seems to correlate to smalt-rich areas and the exposed cream colored underlying lead white and smalt layer (Fig. 11c; f). As mapped by RIS and correlated with visual observations under the microscope, intact blue smalt particles are still visible, but they are often present in a brownish matrix. The brownish matrix, as proposed by Spring, relates to K-soaps^74^. The presence of degradation products, such as that formed in situ, such as palmierite, weddellite and discolored smalt, likely contributes to the brownish appearance and the formation of surface crusts. (Fig. 10f). In some parts of the composition, such as the partisan, the cuffed feathers and the details around the gorget, a smalt-containing paint was used that does not contain lead white. This paint looks visually more discolored and degraded, resulting in a monochrome (dark) brown translucent appearance and loss of detail and modeling of the paint.

**Fig. 12:**
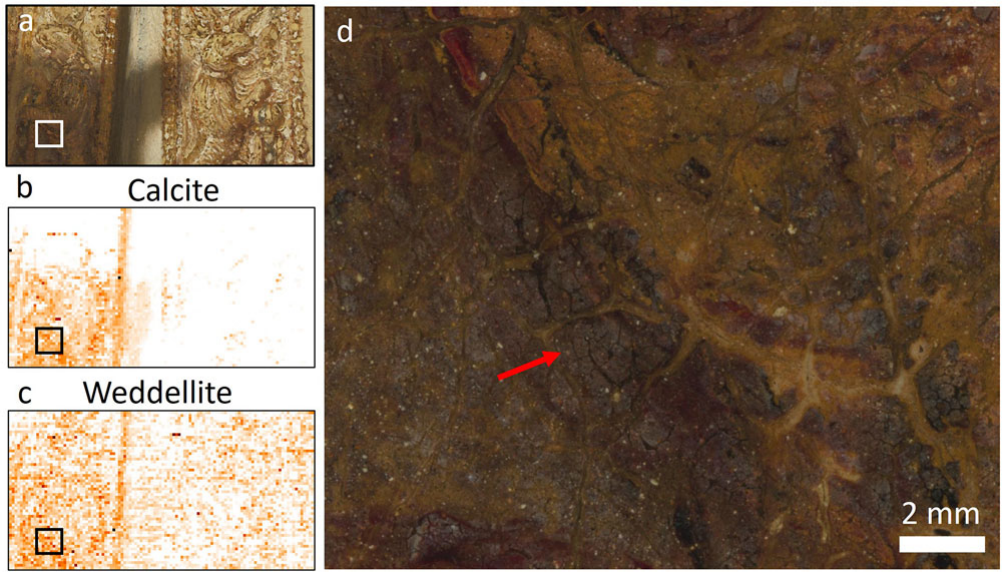
Whitish haze correlated to red lake and bone black rich areas. **a** Visible image of the embroidered border at the height of the lions, Distribution images obtained from MA-XRPD in reflection mode, based on the intensity scaling parameter, for **b** calcite and **c** weddellite. **d** Detail of the 5 µm resolution image, of which the location is indicated with a box in (**a**-**c**), where a whitish haze is visible (red arrow) in the shadows of the embroidery especially correlated to red lake and bone black rich areas, and with the presence of calcite and the calcium oxalate weddellite.

Weddellite (CaC_2_O_4_·2H_2_O), calcium oxalate dihydrate, was also detected by MA-XRPD and is strongly associated with the calcium or calcite-rich areas of the costume. The formation of this secondary product seems to especially visually impact the red lake and bone black paint mixtures (Fig. 12a-d). A whitish haze or even a thick white crust can be observed in those areas where weddellite is mapped (Fig. 12d). The formation of weddellite in paintings, as proposed by de Meyer et al., is likely induced by the degradation of organic paint compounds such as the organic dye of the lake pigments, the binder and varnishes, and degraded smalt oil paint which all release oxalate acids that consequently precipitate with calcium salts^74,106,109,110^.

Although the costume of Van Ruytenburch with its marvelous impastos and details still evokes fascination from the public and scholars as it appears today, from the above observations we can infer that chemical transformations of the original pigments have compromised the appearance since the time Rembrandt painted *The Night Watch*. The paint components that appear to be most affected by these degradation processes are pigments such as the arsenic sulfide pigments, red lake, bone black and smalt, all responsible for the deep shadow modeling. The fading, blanching or browning of these regions perturb the intended harmoniously balanced nuances of the light in shadow modeling. Rembrandt’s meticulously build-up of light-shadow contrasts were therefore altered and have a more subdued appearance today than what he originally intended.

The combined results from RIS-VNIR, MA-XRF and MA-XRPD enabled us not only to map the pigments that were used to paint Van Ruytenburch’s costume but also to study Rembrandt’s painting technique with higher chemical specificity than ever before. This has revealed the systematic use and grouping of pigments to build up the illuminated and shadowed modeling, according to the desired lighting conditions. Additionally, the technique could be studied and linked directly to a passage on Rembrandt’s *chiaroscuro* technique written by his pupil, Samuel van Hoogstraten. By mapping pigments on the macroscale, it has become clear that Rembrandt, through acute observation and skill, paid meticulous attention to the rendering of the different materials in the buff jerkin of the costume and their optical properties such as the warm orange golden glow reflection of the gold threads, painted with an arsenic-based orange pigment mixture. With RIS-VNIR we were able to demonstrate that Rembrandt utilized optical tricks, what is now known as the simultaneous contrast effect, to manipulate the perception of light and shadow. This shows that Rembrandt was not only aware of contemporary ideas regarding perception theories, but also brought them into practice to render a convincing natural transition of light and shadow. In conjunction with OCT, cross-section analysis and surface microscopy, the lead white and smalt-containing underpaint, which was used to create impasto paint and texture, could be characterized. This layer was used in the early steps of the paint process to imitate the material surface textures and contributes to the majority of the textured appearance. In addition, MA-XRPD imaging allowed mapping of degradation products related to the original pigments, enabling a better understanding of the original intent of the painter, hence the implication these secondary products have on the current appearance of the costume. The degradation and fading of specific pigments used for the shadows, such as arsenic sulfides, red lakes, smalt, and bone black, decreased the light and color contrasts, which were initially intended to be more vibrant. Overall, the results have also provided valuable insight to guide the treatment phase. For instance, arsenic species are highly sensitive and require cleaning methods with a short retention time, while avoiding (water-based) cleaning agents to prevent mobilization and further migration^100^. Additionally, the mapping of both original materials and degradation products in their current state serves as a reference for future research. It enables long-term monitoring of material transformations—such as pigment alteration and associated color changes—thereby supporting informed preservation strategies over time.

## Supplementary information

Supplementary information

## Data Availability

All data needed to evaluate the conclusions in the paper are present in the paper and/or the Supporting information file. Additional data related to this paper may be requested from the authors.

## Abbreviations

MA-XRF: macroscopic X-ray fluorescence
MA-XRPD: macroscopic X-ray powder diffraction
RIS-VNIR: reflectance imaging spectroscopy—visible to near infrared spectral range
SEM-EDX: scanning electron microscopy energy dispersive X-ray spectroscopy
µ-XRPD: microscopic X-ray powder diffraction
SR: synchrotron
