# Supplementary material for: Illuminating Rembrandt’s Chiaroscuro in The Night Watch: the painting process of Van Ruytenburch’s costume
Source: NPJ Herit Sci. 2025 Aug 18;13(1):406. doi: 10.1038/s40494-025-01874-w (PMC12360951; doi:10.1038/s40494-025-01874-w)
Supplement: Supplementary file 1 — Supplementary information [file 40494_2025_1874_MOESM1_ESM.pdf]

Supporting Information

Title: Illuminating Rembrandt's *Chiaroscuro* in *The Night Watch*: The Painting Process of Van Ruytenburch's Costume

Authors: Nouchka De Keyser[1,2,3,4]*, Annelies van Loon[2,5], Francesca Gabrieli[2], Frederik Vanmeert[2,3] Fréderique T. H. Broers[1,2,3,6], Petria Noble[2], Steven De Meyer[3,7], Arthur Gestels[3,8], Victor Gonzalez[2,9], Mitra Almasian[10], Inez van der Werf[11], Erma Hermens[12], Koen Janssens[2,3,4], Katrien Keune[1,2]

Affiliations:

(1)University of Amsterdam, Van 't Hoff Institute for Molecular Sciences,1090GD Amsterdam, The Netherlands
(2)Rijksmuseum, Conservation & Science, Museumstraat 1, 1070 DN Amsterdam, The Netherlands
(3)University of Antwerp, Department of Physics, AXIS, Groenenborgerlaan 171, 2020 Antwerp, Belgium
(4)University of Antwerp, Faculty of Design Sciences, ARCHES, Mutsaardstraat 31, 2000 Antwerp, Belgium
(5)Mauritshuis, Collection and Science, Plein 29, 2511 CS The Hague, The Netherlands
(6)Utrecht University, Inorganic Chemistry and Catalysis, Institute for Sustainable and Circular Chemistry, Universiteitsweg 99,3584CG Utrecht, The Netherlands
(7)Royal Institute for Cultural Heritage (KIK-IRPA), Paintings Laboratory, Jubelpark 1, 1000 Brussels, Belgium
(8)University of Antwerp, Department Electromechanics, Research Group InViLab, University of Antwerp, Groenenborgerlaan 171, 2020 Antwerp, Belgium
(9)Université Paris-Saclay, ENS Paris-Saclay, CNRS, PPSM, 91190, Gif-sur-Yvette, France. 9)
(10)Biomedical Engineering and Physics, Amsterdam UMC, University of Amsterdam, Amsterdam, The Netherlands
(11)Cultural Heritage Agency of the Netherlands (RCE), Hobbemastraat 22, 1071 ZC, Amsterdam, the Netherlands.
(12)Hamilton-Kerr Institute and Conservation and Science Division, Fitzwilliam Museum, University of Cambridge, Trumpington Street, Cambridge, CB2 1RB, United Kingdom

*Corresponding author. Email: N.de.Keyser@rijksmuseum.nl

Keywords **– Rembrandt, The Night Watch, Chiaroscuro, Pigment palette, MA-XRF, RIS VNIR, mapping, painting technique**

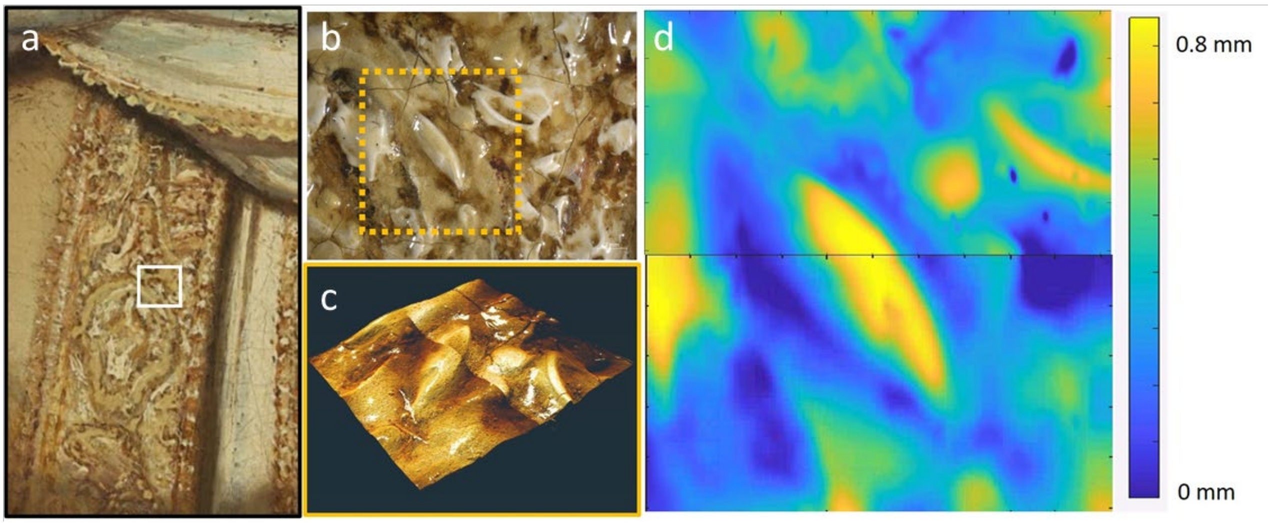

Figure S1. OCT heightmap of an impasto in Van Ruytenburch's embroidered buff jerkin. (a) Detail of Van Ruytenburch's costume (b) stereomicroscopic detail at 0.63x magnification (location indicated with a white box in a), (c) OCT-mapped area, indicated by a yellow dotted box in (b). (d) corresponding heightmap of the OCT-mapped area.

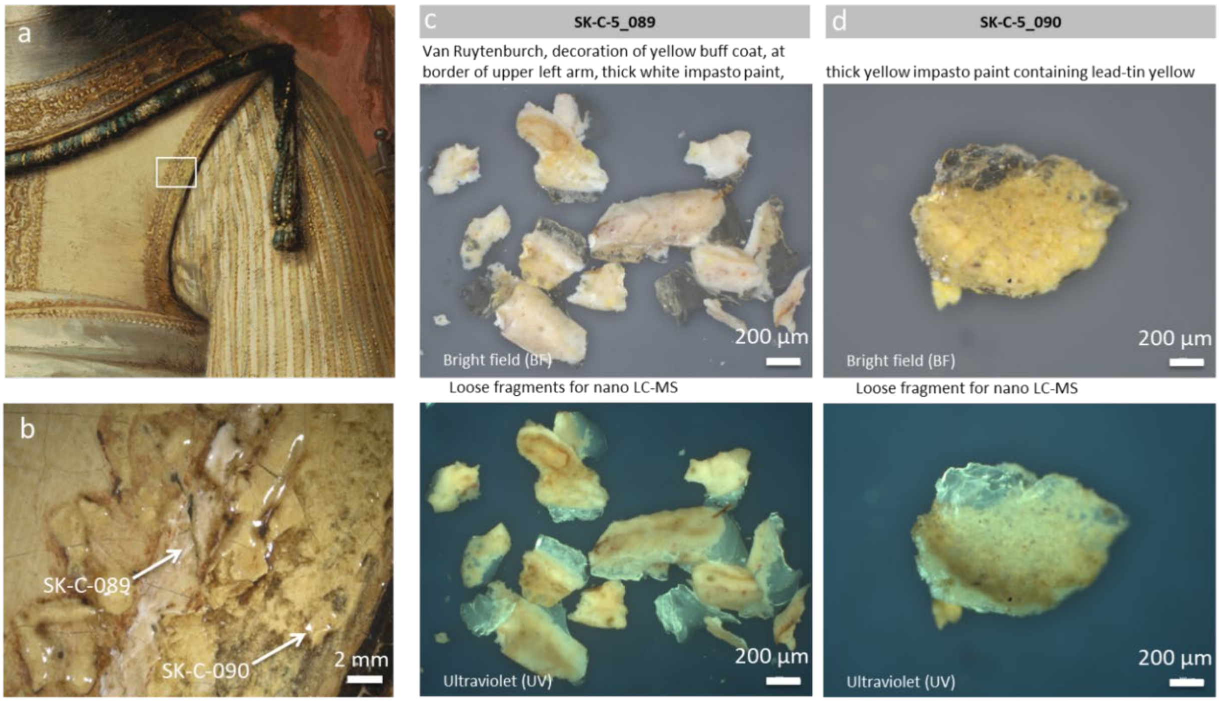

Figure S2. Sample information for proteomic research with nano LC-MS (a) Detail of Van Ruytenburch's costume indicating with a white box the area of sample location **(b)** microphotograph taken with the stereomicroscope of the sample locations for SK-C-5_089 and SK-C-5_090, indicated with white arrows**. (c)** Samples SK-C-089 photographed under the light microscope in bright field (BF) and Ultraviolet (UV) light conditions. **(d)** Sample SK-C-090 photographed under the light microscope in bright field (BF) and Ultraviolet (UV) light conditions

SAMPLE#	OIL (VOL%)	EGG YOLK (VOL%)	EGG WHITE (VOL%)	PVC (PIGMENT VOLUME CONCENTRATION)
**1**	95 (raw)	5	0	35
**2**	95 (Commercial Boiled)	5	0	Max
**3**	95 (PbO Boiled)	5	0	55 (Max)
**4**	95 (raw)	0	5	35
**5**	83 (Commercial Boiled)	17	0	35 (Max)
**6**	70 (raw)	30	0	37 (Max)
**7**	100 (PbO Boiled)	0	0	54.2 (Max)

Table S1. Recipe of the Oil/ Yolk/ Egg white and lead white reconstructions made by Rosen Gramatikov, Master student at the Rijksmuseum, Sept 2020 - July 2021. Samples from these reconstructions were used to validate the methodology of nano LCMS for samples SK-C-5_089 and SK-C-5_090**.**

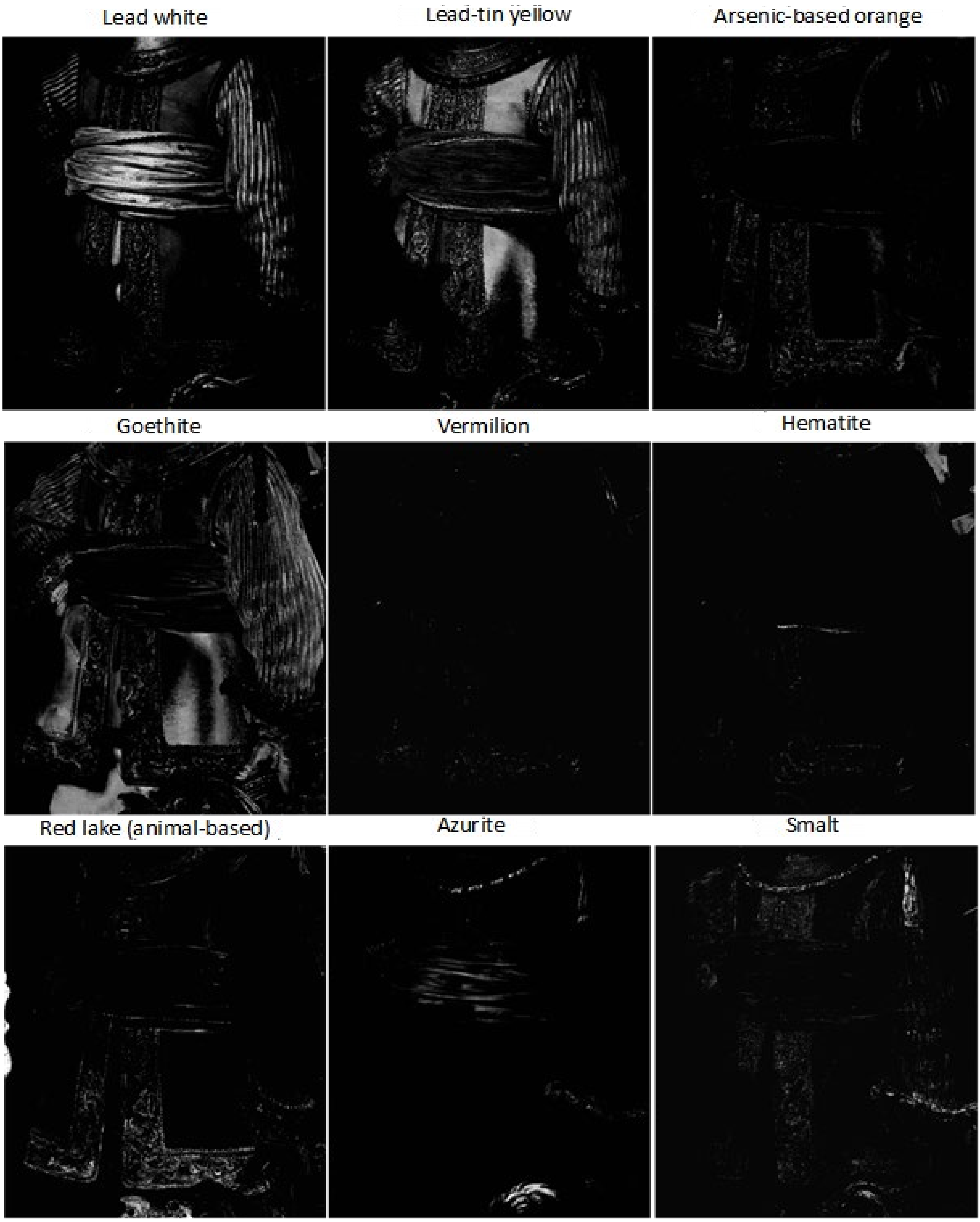

Figure S3. SAM-maps obtained from the first derivative reflectance spectral endmembers for lead white, lead tin yellow, an arsenic-based orange pigment, goethite, vermilion, hematite, an animal-based red lake, azurite and smalt.

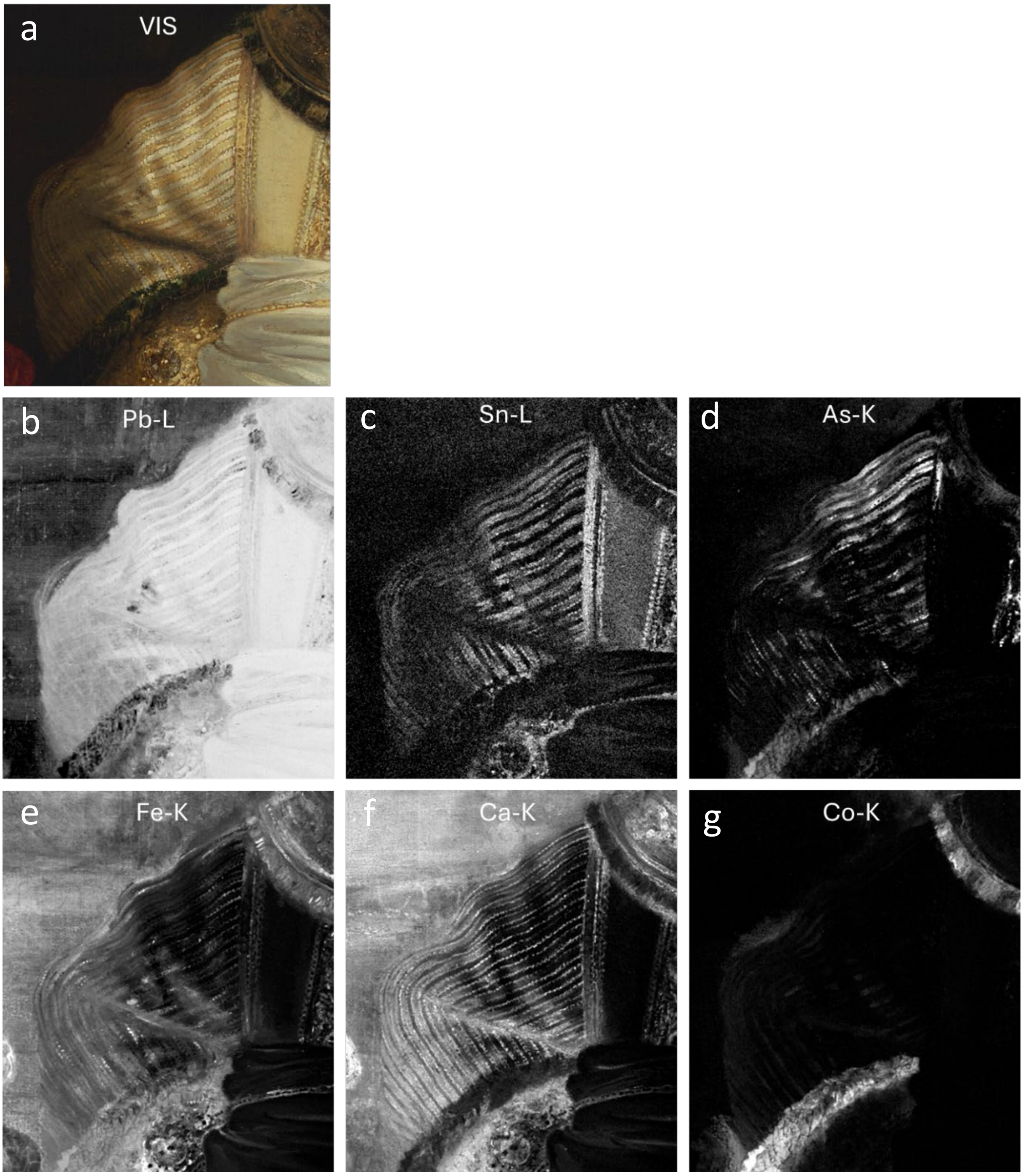

Figure S4. (a) Detail of Van Ruytenburch's sleeve with corresponding MA-XRF maps for **(b)** lead, **(c)** tin, **(d)** arsenic, **(e)** iron, **(f)** calcium and **(g)** cobalt.

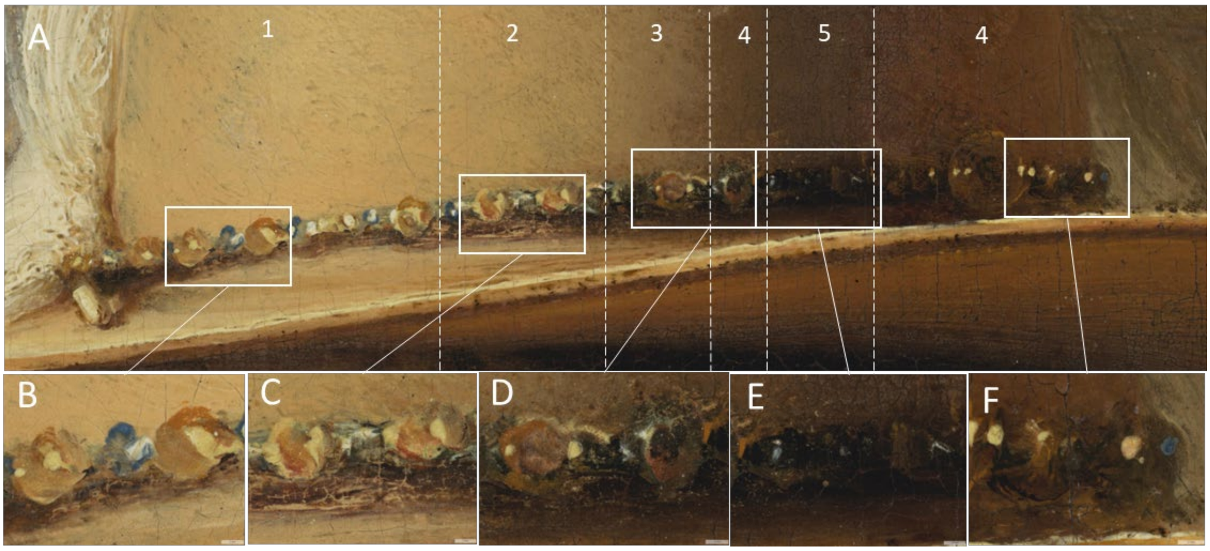

Figure S5. Light to dark grades discussed by Samuel van Hoogstraten (1627-1678) in his fifth chapter on light and shadow in *Inleyding tot de hooge schoole der schilderkunst: anders de zichtbaere werelt* (1678). (a) Visible light image of the pearled hat decoration of Ruytenburch with details of the pearls (b-f) rendered by Rembrandt according to the grades of illumination as described by Hoogstraten: 1: brightest (maximum) lights, 2: grazing tones, half-lit, 3: as only a quarter illuminated, we put the usual reflections and scumbles and transparencies, and everything that can be recognized in the shadows, 4: shadows which are nevertheless affected by some half-light, 5: hollow depths, those bereft of all light

Regel van dagen en schaduwen te schikken[1]
‘*’Daerom beveele ik u niet te veel met lichten en schaduwen door een te haspelen, maer de zelve bequamelijk in groepen te vereenigen; laet uwe sterkste lichten met minder lichten minlijk verzelt zijn, ik verzeeker u, dat ze te heerlijker zullen uitblinken; laet uwe diepste donkerheden met klaere bruintens omringt zijn, op dat ze met te meerder gewelt de kracht van het licht mogen doen afsteken. Rembrandt heeft deeze deugt hoog in top gevoert, en was volleert in 't wel byeenvoegen van bevriende verwen.*”

[1] Hoogstraten S van. Inleyding tot de hooge schoole der schilderkonst: anders de zichtbaere werelt; verdeelt in negen leerwinkels, yder bestiert door eene der zanggodinnen; ten hoogsten noodzakelijk, tot onderwijs, voor alle die deeze edele, vrye, en hooge konst oeffenen, of met yver zoeken te leeren, of anders eenigzins beminnen. fotografische herdruk. Davaco; 1969, p 306.

Rules to arrange the days and shadows
‘’I therefore recommend you not to mix up lights and shadows too much, but to combine them properly in groups; let your strong lights be gently accompanied by lesser lights, and I assure you that they will shine all the more beautifully; let your deepest darks be surrounded by lighter darks, so that they will make the power of the light standout all the more powerfully. Rembrandt developed this virtue to a high degree, and he was a master in properly combining related colours”[2]
[2] Wetering E van de. Rembrandt: The Painter at Work. Amsterdam University Press; 1997
